# Supplementary material for: N-AS-triggered SPMs are direct regulators of microglia in a model of Alzheimer’s disease
Source: Nat Commun. 2020 May 12;11:2358. doi: 10.1038/s41467-020-16080-4 (PMC7217877; doi:10.1038/s41467-020-16080-4)
Supplement: Supplementary file 1 — Supplementary Information [file 41467_2020_16080_MOESM1_ESM.pdf]

Supplementary Information

N-AS-triggered SPMs are direct regulators of microglia in a model of Alzheimer disease

Ju Youn Lee,[1,2,3,10] Seung Hoon Han,[1,2,3,10] Min Hee Park,[1,2,3] Im-Sook Song,[4] Min-Koo Choi,[5] Eunsoo Yu,[6] Cheol-Min Park,[6] Hee-Jin Kim,[7] Seung Hyun Kim,[7] Edward H. Schuchman,[8] Hee Kyung Jin,[1,9,*] Jae-sung Bae[1,2,3,*]

Supplementary discussion

Acetyl-CoA interacts with the ATP binding pocket of SphK1

In order to gain insights into the mechanism of the acetyltransferase activity of SphK1, we began by building a model of acetyl-CoA bound to SphK1 based on the crystal structure of the SphK1-ATP complex[1,2]. Docking simulation suggested that acetyl-CoA occupies an extended cleft between the N-terminal and C-terminal domains, which is also known as the nucleotide binding site of SphK1[1,2] (Supplementary Fig. 1a). Detailed analysis further suggested that acetyl-CoA was held in place by several key interactions involving arginine residues in the active site, in particular arginine 56 (R56) and arginine 57 (R57), via interaction of the adenine moiety by hydrophobic interaction and hydrogen bonding, respectively. Furthermore, there were extended interactions of the tail portion of acetyl-CoA with the protein through the hydrogen bonding of the α-phosphate and pantothenic amide moiety with arginine 24 (R24) and arginine 185 (R185), respectively, along with a magnesium ion bridging the two groups of acetyl-CoA, the homoalanine carbonyl and thioester groups, providing tight substrate binding (Supplementary Fig. 1b, c). These binding interactions identified in our model turn out to be consistent with those in the crystal structure of the SphK1-ATP complex, suggesting that acetyl-CoA might be compatible with the ATP binding pocket of SphK1 (Supplementary Fig. 1d).

To further investigate the competitive relationship of acetyl-CoA and ATP in the SphK1 binding site, we examined S1P production by SphK1 in the presence of acetyl-CoA. Acetyl-CoA inhibited S1P formation in a concentration dependent fashion (Supplementary Fig. 1e). We also conversely assessed whether ATP inhibited acetyl-CoA binding to SphK1. Incorporation of acetyl-CoA into SphK1 was decreased with increasing concentrations of ATP, yielding K_M values of 24.51, 35.21, and 39.01 μM at 10, 100, and 1000 μM ATP, respectively. The K_{cat} and enzymatic efficiency (K_{cat}/K_M) were also decreased in a concentration dependent manner by ATP, indicating that acetyl-CoA binding to SphK1 was inhibited by ATP (Supplementary Fig. 1f). Moreover, to test for the importance of residues R56, R57, R24, and R185 in the putative acetyl-CoA binding pocket of SphK1, the residues were mutated into alanine residues, R56A, R57A, R24A, and R185A, respectively. All mutants, especially R56A, have higher K_M values and reduced enzymatic efficiency compared with the control (Supplementary Fig. 1g). Overall, these results suggested that acetyl-CoA and ATP had likely occupied the same site in SphK1, and that residues R56, R57, R24, and R185 played critical roles in the acetyl-CoA binding pocket of SphK1.

Previously, our studies had revealed that acetyl-CoA bound to SphK1 with sphingosine, which led to COX2 acetylation[3]. To further investigate the mechanism of

SphK1-mediated COX2 acetylation, we incubated COX2 with SphK1, sphingosine, and [^{14}C] acetyl-CoA in the presence of ATP as a competitor of acetyl-CoA, and then COX2 acetylation was analyzed. The results showed that SphK1-mediated COX2 acetylation was inhibited in a concentration dependent manner by ATP (Supplementary Fig. 1h). Moreover, COX2 acetylation was also significantly decreased in mutants of the acetyl-CoA binding pocket within SphK1 (Supplementary Fig. 1i). These results demonstrated that acetyl-CoA binding to SphK1 is important for SphK1-mediated COX2 acetylation.

N-AS is generated via acetyl-CoA and sphingosine binding to SphK1

As described above, both acetyl-CoA and sphingosine were required for SphK1-mediated COX2 acetylation, suggesting that sphingosine may participate in this catalytic reaction[3]. To examine the role of sphingosine and acetyl-CoA in SphK1-mediated COX2 acetylation, we performed a docking simulation on the ternary complex of acetyl-CoA, sphingosine, and SphK1. As shown in Supplementary Fig. 2a, the docked model predicted close proximity between sphingosine and the acetyl group of acetyl-CoA, facilitating facile acetyl transfer reaction in SphK1. This docking simulation also suggested the ability of SphK1 to promote transfer of the acetyl group of acetyl-CoA to sphingosine, and the possible existence of three potential isomers of acetylated sphingosine, 1-O-AS,

3-O-AS or N-AS, for the acetyl transfer to COX2. To shed further light on the identity of the isomer responsible for the acetyl transfer to COX2, we examined the stability of each isomer under physiological conditions. We observed rapid isomerization of both 1- and 3-O-AS to N-AS in less than 10 min when incubated in phosphate buffer, pH 7.4 (Supplementary Fig. 2b). This strongly suggested that the species that acetylates COX2 is N-AS, even though we cannot completely rule out the remote possibility of the transient formation of O-AS in the active site of SphK1 followed by rapid isomerization to N-AS before binding to the COX2 active site.

Although N-AS is also known as C2-ceramide and its treatment exerts anti-tumor effects by inducing programmed apoptosis and necrosis in cancer cells[4,5], the biosynthetic pathway of N-AS have not been identified. To investigate whether N-AS, an intermediate candidate for COX2 acetylation, was produced by acetyl-CoA and sphingosine in SphK1, purified SphK1 was incubated with acetyl-CoA and sphingosine N-AS was synthesized by this reaction, further suggesting that N-AS might be the intermediate for SphK1-mediated COX2 acetylation (Supplementary Fig. 2c). Mechanistically, we propose that the acetylation of sphingosine occurs via general base catalysis, in which the ammonium group of sphingosine held in place by the phenylalanine 192 (F192) cation-π interaction in the SphK1 active site was turned into a

nucleophilic amine via deprotonation by aspartic acid 178 (D178), resulting in the acetyl group transfer from acetyl-CoA (Supplementary Fig. 2d). To corroborate the role of D178 and F192 in catalysis, we generated alanine substitution mutants for D178 and F192, and followed the formation of N-AS. Although wild type (WT) SphK1 produced more N-AS with increasing concentrations of acetyl-CoA, the D178A and F192A mutants produced less N-AS compared with control, indicating that D178 and F192 played a critical role in the acetyl transfer step (Supplementary Fig. 2e). Taking all of these data together, we identified the generation of N-AS by SphK1 via its interaction with acetyl-CoA and sphingosine. We also established D178 and F192 as catalytically critical residues for the production of N-AS, and found that N-AS is an intermediate for SphK1-mediated COX2 acetylation.

Pharmacokinetic properties of N-AS

Before investigating the relationship between N-AS generation and pathogenesis in an AD mouse model, we first examined the PK profile of N-AS ADME (absorption, distribution, metabolism and excretion) using in vitro assays, and found that about 50-60 % of the compound remained after incubation for 30 min with human liver microsomes (Supplementary Fig. 3a). The drug-drug interaction potential of N-AS also was tested.

Probe substrates for seven major cytochrome P450 isozymes (1A2, 2B6, 2C9, 2C19, 2D6, 2E1, and 3A4) were incubated with various concentrations of N-AS (0-100 μM) in the presence of the human liver microsomes. Inhibition of the seven major cytochrome P450 enzymes by N-AS was negligible; for example, more than 80 % of metabolic activity for these enzymes was retained in the presence of 100 μM N-AS (Supplementary Fig. 3b). Next, a single-dose PK profile and tissue distribution of N-AS in C57BL/6 mice were determined after intravenous (i.v.) (1 mg kg^{-1}) and oral (p.o.) (10 mg kg^{-1}) administration of N-AS. The plasma and brain concentration-time curves and the PK parameters of N-AS after its administration are presented in Supplementary Fig. 3c-f. Although the bioavailability (BA) was low, N-AS was quickly distributed into the brain and the elimination half-life was slow compared with that of plasma. At 24 hours post administration, brain showed the highest concentration of N-AS, with little to no detectable amounts in the liver, kidney, and heart (Supplementary Fig. 3g). These results suggested that N-AS may have favorable therapeutic efficacy in brain disorders with chronic inflammation.

N-AS generation is also reduced in Aβ-treated human microglia

In order to assess the clinical importance of N-AS in microglia, we induced an AD environment in human microglia using Aβ, and checked the generation and roles of N-AS. N-AS generation was decreased in Aβ-treated microglia compared with microglia without treatment, leading to reduced acetyl-S565 COX2 and SPM secretion. These were restored by acetyl-CoA treatment, suggesting that deficient acetyl-CoA induced by Aβ led to reduced N-AS generation, leading to microglial dysfunction in the AD environment (Supplementary Fig. 6a-d). We also explored whether the observed effects of N-AS treatment in the mouse models were paralleled by similar alterations in human microglia. When N-AS was used to treat Aβ-affected microglia, S565 acetylation of COX2 and SPM secretion were restored, leading to enhanced phagocytic capacity (Supplementary Fig. 6 and Supplementary Videos 1-6). Further, we checked whether N-AS-triggered SPMs directly mediate the effects seen after the administration of N-AS. To block SPM production, we used the 5-LOX inhibitor, zileuton[6], in the presence of N-AS. When zileuton was incubated with N-AS in Aβ-treated human microglia, no significant differences in N-AS-induced COX2 acetylation were observed compared with in N-AS and Aβ-treated microglia without zileuton (Supplementary Fig. 6b, c). However, SPM secretion was significantly reduced with the treatment of zileuton, indicating that zileuton specifically inhibited SPM secretion, rather than affecting COX2 acetylation by N-AS

(Supplementary Fig. 6d and Supplementary Table 3). Interestingly, the inhibition of SPM secretion by zileuton in the presence of N-AS blocked the restoration of phagocytic capacity in Aβ-treated microglia via N-AS, indicating that N-AS-triggered SPMs directly mediate the positive effects in microglia (Supplementary Fig. 6e, f). These results suggested N-AS-triggered SPMs were responsible for direct regulation of microglia including phagocytosis in a human AD environment, supporting the potential clinical effects in AD patients.

Supplementary Table Legends

Supplementary Table 1. Comparison of product profiles for AA, EPA, and DHA incubated with huCOX2, aspirin-acetylated huCOX2, and N-AS-acetylated huCOX2.

	**Naïve**	**Aspirin**	**N-AS**
**Fatty Acids**		**Product (%)**	
**AA bioactive metabolome**			
PGH_2	75.5±0.40	72.3±0.68*	69.7±1.08***
11-HETE	12.3±0.22	13.8±0.41	13.1±0.55
15-HETE	7.8±0.22	9.5±0.22*	13.3±0.59****####
15R-LXA_4	3.8±0.22	4.2±0.18	4.8±0.23*
**EPA bioactive metabolome**			
11-HEPE	30.7±0.25	27.3±1.40*	26.8±0.41*
15-HEPE	5.6±0.23	5.8±0.06	5.8±0.09
18-HEPE	61.4±0.10	61.3±0.32	64.0±0.25****####
RvE1	2.1±0.11	3.1±0.25**	3.3±0.09***
**DHA bioactive metabolome**			
13-HDHA	8.0±0.32	5.0±0.15****	3.6±0.09****
17-HDHA	91.8±0.30	94.6±0.22****	95.8±0.19****##
RvD1	0.19±0.03	0.4±0.01*	0.53±0.09**
RvD2	ND	ND	ND
RvD3	ND	ND	ND

PGH_2 prostaglandin H2, PGE_2 prostaglandin E2, 11-HETE 11-hydroxy-5Z,8Z,12E,14Z-eicosatetraenoic acid , 15-HETE 15-hydroxy-5Z,8Z,11Z,13E-eicosatetraenoic acid, 11-HEPE 11-hydroxy-5Z,8Z,12E,14Z,17Z-eicosapentaenoic acid, 15-HEPE 15-hydroxy-5Z,8Z,11Z,13E,17Z-eicosapentaenoic acid, 18-HEPE 18-hydroxy-5Z,8Z,11Z,14Z,16E-eicosapentaenoic acid, 13-HDHA 13-hydroxy-4Z,7Z,10Z,14E,16Z,19Z-docosahexaenoic acid, 17-HDHA 17-hydroxy-4Z,7Z,10Z,13Z,15E,19Z-docosahexaenoic acid. n = 6 independent experiments per group. All data are expressed as mean±s.e.m. One-way analysis of variance, Tukey's post hoc test. *$P < 0.05$, **$P < 0.01$, ***$P < 0.001$, ****$P < 0.0001$ versus naive. ##$P < 0.01$, ####$P < 0.0001$ versus aspirin.

Supplementary Table 2: Microglia lipid mediator (LM)-SPM profiles from WT, APP/PS1, and APP/PS1 treated with N-AS.

			LM in mouse microglia (pg/1*10^{6}cell)		
**Bioactive metabolome**	**Q1**	**Q3**	**WT**	**APP/PS1**	**APP/PS1/N-AS**
**AA bioactive metabolome**					
AA	303.1	259.1	37989.3±2423.5	40437.6±2399.6	39563.5±824.8
PGH_2/PGE_2	351	271	3305.4±575.3	3174.1±532.9	3862.0±266.4
TxB_2	369	169	ND	ND	ND
11-HETE	319	167	7959.3±1223.2	6899.2±675.0	8870.6±557.1
LTB_4	335	195	838.8±168.3	719.4±51.5	876.5±71.9
15-HETE	319	219	32247.6±5423.3	21677.7±301.8*	33117.1±1929.6$^{\#}$
15R-LXA_4	351	115	4528.3±214.7	3116.4±28.9**	4378.2±241.8$^{\#\#}$
**EPA bioactive metabolome**					
EPA	301.1	257.1	341.3±8.4	380.1±24.9	406.6±21.1
PGE_3/PGD_3	349	269	2050.3±292.4	1609.8±338.6	2590.3±243.8
TxB_3	367	169	1177.8±95.7	999.1±98.0	1209.9±70.0
11-HEPE	317	167	7175.8±506.4	6259.4±422.7	8016.0±190.1
15-HEPE	317	219	4871.9±317.7	4158.0±278.9	5286.6±214.8
18-HEPE	317	215	26502.7±530.4	18149.4±691.1*	31965.9±2934.1$^{\#\#}$
RvE1	349	195	175.2±36.8	64.9±4.4*	151.8±16.3$^{\#}$
**DHA bioactive metabolome**					
DHA	327.2	283.1	32883.5±3044.1	33346.1±1826.4	37384.7±1351.7
13-HDHA	343	193	6217.1±498.2	6164.8±489.6	6824.5±237.7
17-HDHA	343	281	70190±4619.2	38935.2±1481.9**	86224.5±10404.3$^{\#\#}$
RvD1	375	141	116.0±19.1	61.3±6.9*	112.4±10.9$^{\#}$
RvD2	375	277	ND	ND	ND
RvD3	375	147	ND	ND	ND

PGE_2 prostaglandin E2, LTB_4 leukotriene B4, TxB2 thromboxane B2, 15R-LxA4 15R-lipoxin A4, PGE_3 prostaglandin E3, PGD_3 prostaglandin D3, TxB_3 thromboxane B3, RvE1 resolvin E1, RvD1 resolvin D1, RvD2 resolvin D2, RvD3 resolvin D3. Microglia were isolated from WT, APP/PS1 and APP/PS1 treated with N-AS (n = 6 mice per group). All data analysis was done at 9-mo-old mice. All data are expressed as mean±s.e.m. One-way analysis of variance, Tukey's post hoc test. *$P < 0.05$, **$P < 0.01$ versus WT. $^{\#}P < 0.05$, $^{\#\#}P < 0.01$ versus APP/PS1.

Supplementary Table 3: Human Microglia LM-SPM profiles from Control treated with N-AS, acetyl-CoA or Zil in presence of Aβ 50μM or not.

			LM in human microglia (ng/ml*10^6cell)						
**Bioactive metabolome**	**Q1**	**Q3**	**Control**	**Control + N-AS**	**Control + Zil + N-AS**	**Aβ**	**Aβ + Acetyl-CoA**	**Aβ + N-AS**	**Aβ + Zil + N-AS**
**AA bioactive metabolome**									
PGH_2/PGE_2	351	271	116.5±1.1	105.8±1.1	79.5±0.9^{***++}	78.7±0.8**	103.1±1.0$^{\#\#}$	95.7±0.9$^{\#\#}$	76.0±1.0$^{**€}$
TxB_2	369	169	ND	ND	ND	ND	ND	ND	ND
11-HETE	319	167	168.5±1.8	161.8±0.9	162.5±0.2	174.9±2.0	151.2±0.5	148.4±1.9	144.4±0.9
LTB_4	335	195	ND	ND	ND	ND	ND	ND	ND
15-HETE	319	219	647.1±7.0	625.5±6.8	610.2±4.4	564.3±4.4**	609.4±1.9$^{\#\#}$	625.4±1.7$^{\#\#}$	612.5±2.7$^{\#\#}$
15R-LXA_4	351	115	31.8±0.4	28.7±0.3	0.03±0.0^{***+++}	0.03±0.0**	29.6±0.3$^{\#\#}$	19.8±0.2$^{\#\#}$	0.02±0.0$^{***€€€}$
**EPA bioactive metabolome**									
PGE_3/PGD_3	349	269	142.9±2.2	113.1±1.6	130.0±1.9	163.9±2.5	158.7±2.5	132.9±2.0	146.7±0.6
TxB_3	367	169	ND	ND	ND	ND	ND	ND	ND
11-HEPE	317	167	484.4±5.8	451.8±4.6	483.1±2.4	490.2±2.4	467.8±4.5	371.7±4.0	480.6±2.3
15-HEPE	317	219	298.9±5.0	284.1±4.0	273.7±4.2	272.1±7.3	297.5±3.8	237.9±3.7	268.2±2.1
18-HEPE	317	215	1729.2±24.1	1636.9±19.7	1650.0±7.6	1515.3±10.4**	1881.1±22.9$^{\#\#}$	1763.6±30.4$^{\#\#}$	1668.4±4.3$^{\#\#}$
RvE1	349	195	22.3±0.3	18.5±0.4	0.01±0.0^{***+++}	0.01±0.0***	14.5±0.2$^{\#\#\#}$	8.6±0.3$^{\#\#\#}$	0.01±0.0$^{***€€€}$
**DHA bioactive metabolome**									
13-HDHA	343	193	146.6±2.1	133.9±2.2	137.6±1.6	128.2±1.8	147.4±1.0	133.0±1.8	140.8±2.9
17-HDHA	343	281	693.3±8.5	633.8±9.2	642.3±12.4	561.8±2.7**	720.8±5.3$^{\#\#}$	707.6±8.2$^{\#\#}$	705.4±6.0$^{\#\#}$
RvD1	375	141	2.5±0.02	2.36±0.02	0.01±0.0^{***+++}	0.01±0.0***	2.75±0.04$^{\#\#\#}$	2.13±0.02$^{\#\#\#}$	0.01±0.0$^{***€€€}$
RvD2	375	277	ND	ND	ND	ND	ND	ND	ND
RvD3	375	147	ND	ND	ND	ND	ND	ND	ND

Human microglia were treated with N-AS, acetyl-CoA or Zil in presence of Aβ 50 μM or not (n = 6 per group). All data are expressed as mean±s.e.m. One-way analysis of variance, Tukey's post hoc test. *$^{**}P < 0.01$, $^{***}P < 0.001$ versus control. $^{\#\#}P < 0.01$, $^{\#\#\#}P < 0.001$ versus Aβ. $^{+}P < 0.05$, $^{++}P < 0.01$, $^{+++}P < 0.001$ versus control+N-AS. $^{€}P < 0.05$, $^{€€€}P < 0.001$ versus Aβ+N-AS.*

Supplementary Table 4. Sequence of mutagenesis primer pairs

Gene		Sequence
SphK1 R24A	Forward	5’-GAACCCGGCCGGCGGCAAGGGCAAGGCC-3’
	Reverse	5’-CCGCCGGCCGGGTTCAGCAGCACCAGC-3’
SphK1 R56A	Forward	5’-CACTGAGGCACGGAACCACGCGCGGGAGC-3’
	Reverse	5’-TTCCGTGCCTCAGTGAGCATCAGCGTGAAGG-3’
SphK1 R57A	Forward	5’-TGAGCGGGCGAACCACGCGCGGGAGCTGG-3’
	Reverse	5’-TGGTTCGCCCGCTCAGTGAGCATCAGCG-3’
SphK1 R185A	Forward	5’-GAAGTATGCTCGTCTGGGGGAGATGCGC-3’
	Reverse	5’-AGACGAGCATACTTCTCACTCTCTAGGTCCACA-3’
SphK1 D178A	Forward	5’-TGATGTGGCGCTAGAGAGTGAGAAGTATCGGCG-3’
	Reverse	5’-TCTAGCGCCACATCAGCAATGAAGCCCC-3’
SphK1 F192A	Forward	5’-GATGCGCGCGACTCTGGGCACCTTCCTGC-3’
	Reverse	5’-AGAGTCGCGCGCATCTCCCCCAGACG-3’
COX2 S565A	Forward	5’-CTTTACTGCGTTCAGTGTTCCAGATCCAGAGC-3’
	Reverse	5’-CTGAACGCAGTAAAGGGACAGCCCTTCACG-3’
COX2 N181A	Forward	5’-GGGCTCAGCTATGATGTTTGCATTCTTTGCCCAGC-3’
	Reverse	5’-ATCATAGCTGAGCCCTGGGGATCAGG-3’
COX2 T564A	Forward	5’-TCCCTTTGCTTCATTCAGTGTTCCAGATCCAGAGC-3’
	Reverse	5’-AATGAAGCAAAGGGACAGCCCTTCACG-3’
COX2 S567A	Forward	5’-TTCATTCGCTGTTCCAGATCCAGAGCTCA-3’
	Reverse	5’-GGAACAGCGAATGAAGTAAAGGGACAGCC-3’

SphK1, sphingosine kinase 1; COX2, cyclooxygenase 2

Supplementary Table 5. Sequences of Real-time PCR primer pairs

Gene	Forward	Reverse
TNF-α	5'-GATTATGGCTCAGGGTCCAA-3'	5'-GCTCCAGTGAATTCGGAAAG-3'
IL-1β	5'-CCCAAGCAATACCCAAAGAA-3'	5'-GCTTGTGCTCTGCTTGTGAG-3'
IL-6	5'-CCGGAGAGGAGACTTCACAG-3'	5'-TTGCCATTGCACAACTCTTT-3'
iNOS	5'-CACCTGGAACAGCACTCTCT-3'	5'-CTTTGTGCGAAGTGTCAGTG-3'
IL-10	5'-AAGGCCATGAATGAATTTGA-3'	5'-TTCGGAGAGAGGTACAAACG-3'
IL-4	5'-ATCCATTTGCATGATGCTCT-3'	5'-GAGCTGCAGAGACTCTTTCG-3'
TGF-β	5'-TTACCTGGATGGAAGTGGAA-3'	5'-TGTTATGAGGAAGGGGACAA-3'
Arg1	5'-AAGCCAAGGTTAAAGCCACT-3'	5'-CGATTCACCTGAGCTTTGAT-3'
COX2	5'-TAGAATCCAGTCCGGGTACA -3'	5'-ACAAAAGAACCCAGTGTCCA-3'
NEP	5'-GAAATTCAGCCAAAGCAAGC-3'	5'-GATTTCGGCCTGAGGAATAA-3'
MMP9	5'-CCATGTCACTTTCCCTTCAC-3'	5'-CTCACTAGGGCAGAAACCAA-3'
IDE	5'-GAAGACAAACGGGAATACCGTG-3'	5'-CCGCTGAGGACTTGTCTGTG-3'
CD36	5'-TGTACACGGGGATTCCTTTA-3'	5'-TCCTATTGGCCAAGCTATTG -3'
GAPDH	5'-TGAATACGGCTACAGCAACA-3'	5'-AGGCCCCTCCTGTTATTATG-3'

TNF-α, tumor necrosis factor-alpha; IL1β, interleukin 1 beta; IL6, interleukin 6; *iNOS*, inducible nitric oxide synthase; *IL10*, interleukin 10; *IL4*, interleukin 4; *TGFβ*, transforming growth factor beta; *Arg1*, arginase 1; *COX2*, cyclooxygenase 2; *NEP*, neprilysin; *MMP9*, matrix metallopeptidase 9; *IDE*, insulin degrading enzyme; *CD36*, cluster of differentiation 36; *GAPDH*, glyceraldehyde 3-phosphate dehydrogenase.

Supplementary References

1. Wang, Z. et al. Molecular basis of sphingosine kinase 1 substrate recognition and catalysis. *Structure* **21**, 798-809 (2013).
2. Adams, D. R., Pyne, S. & Pyne, N. J. Sphingosine kinases: emerging structure-function insights. *Trends Biochem. Sci.* **41**, 395-409 (2016).
3. Lee, J. Y. et al. Neuronal SphK1 acetylates COX2 and contributes to pathogenesis in a model of Alzheimer's disease. *Nat. Commun.* **9**, 1479 (2018).
4. Jung, J. S., Ahn, Y. H., Moon, B. I. & Kim, H. S. Exogenous C2 ceramide suppresses matrix metalloproteinase gene expression by inhibiting ROS production and MAPK signaling pathways in PMA-stimulated human astroglioma cells. *Int. J. Mol. Sci.***17,** 477 (2016).
5. Zhu, W., Wang, X., Zhou, Y. & Wang, H. C2-ceramide induces cell death and protective autophagy in head and neck squamous cell carcinoma cells. *Int. J. Mol. Sci.* **15**, 3336-3355 (2014).
6. Rossi, A. et al. The 5-lipoxygenase inhibitor, zileuton, suppresses prostaglandin biosynthesis by inhibition of arachidonic acid release in macrophages. Br. J. Pharmacol. **161**, 555-570 (2010).

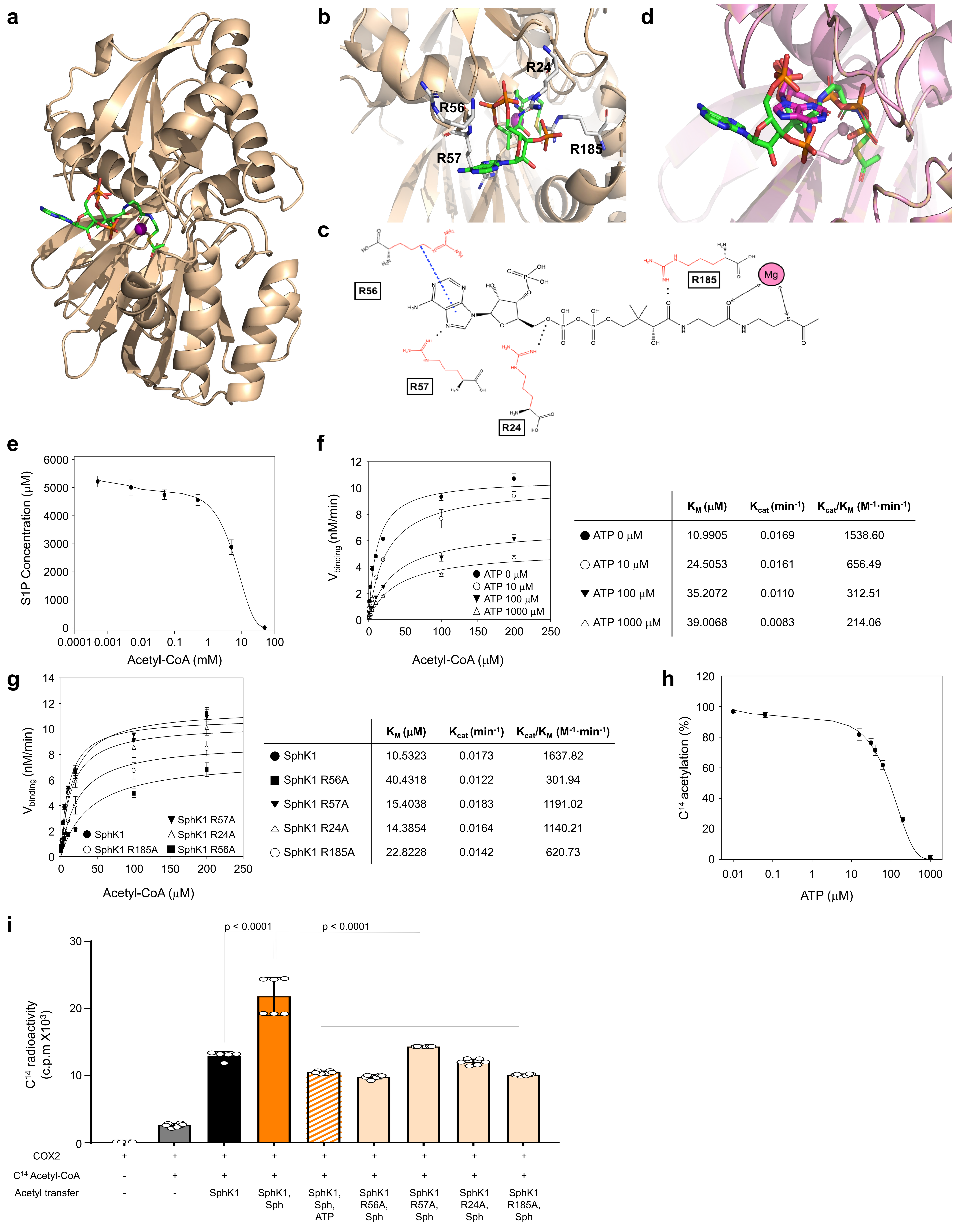

	K_M (μM)	K_{cat} (min^{-1})	K_{cat}/K_M (M^{-1}·min^{-1})
● ATP 0 μM	10.9905	0.0169	1538.60
○ ATP 10 μM	24.5053	0.0161	656.49
▼ ATP 100 μM	35.2072	0.0110	312.51
△ ATP 1000 μM	39.0068	0.0083	214.06

	K_M (μM)	K_{cat} (min^{-1})	K_{cat}/K_M (M^{-1}·min^{-1})
● SphK1	10.5323	0.0173	1637.82
■ SphK1 R56A	40.4318	0.0122	301.94
▼ SphK1 R57A	15.4038	0.0183	1191.02
△ SphK1 R24A	14.3854	0.0164	1140.21
○ SphK1 R185A	22.8228	0.0142	620.73

Supplementary Figure 1: Acetyl-CoA binds the ATP binding site in SphK1. **a,** Docking results of acetyl-CoA to SphK1. Acetyl-CoA bound to the homology model of the cleft formed by N- and C-terminal domains of SphK1 (Mg^{2+} ion denoted by a purple sphere). **b,** Expanded view of the residues proximal to acetyl-CoA within the orthosteric binding site (stick representation for the key residues). **c,** Detailed interaction map between SphK1 and acetyl-CoA. **d,** Overlay of acetyl-CoA and ATP docked into the SphK1 binding site. **e,** Production of S1P in the presence of increasing acetyl-CoA concentration ranging from 0-50 mM using UPLC system (n = 6 independent experiments per group). **f,** Acetyl-CoA binding activity of SphK1 was analyzed by filter binding assay in the presence of 0, 10, 100, and 1000 μM ATP with 100 μM sphingosine. The binding velocity ($V_{binding}$) of [^{3}H] acetyl-CoA to SphK1 was plotted to the acetyl-CoA concentration and the nonlinear regression analysis of the saturated plot yielded the kinetic parameters such as K_{cat} (catalytic constant) and K_M (Michaelis − Menten constant) for acetyl-CoA and SphK1 binding activity (n = 3 independent experiments per group). **g,** Acetyl-CoA binding activity of SphK1 WT, SphK1 R56A, SphK1 R57A, SphK1 R24A, and SphK1 R185A was analyzed by filter binding assay with 100 μM sphingosine (n = 3 independent experiments per group). **h,** Acetylation assay of purified COX2 protein treated with SphK1 and [^{14}C] acetyl-CoA in the presence of 100 μM sphingosine and increasing ATP concentration ranging from 0-1000 μM. The purified COX2 protein incubated in the presence of [^{14}C] acetyl-CoA, sphingosine and ATP for 2 h at 37 °C and then COX2 was analyzed on scintillation counter (n = 3 independent experiments per group). **i,** Acetylation assay of recombinant COX2 treated with SphK1 WT, SphK1 R56A, SphK1 R57A, SphK1 R24A or SphK1 R185A, and [^{14}C] acetyl-CoA in the presence of 100 μM sphingosine (n = 6 independent experiments per group). i, One-way analysis of variance, Tukey's post hoc test. All error bars indicate s.e.m. Source data are provided as a Source Data file.

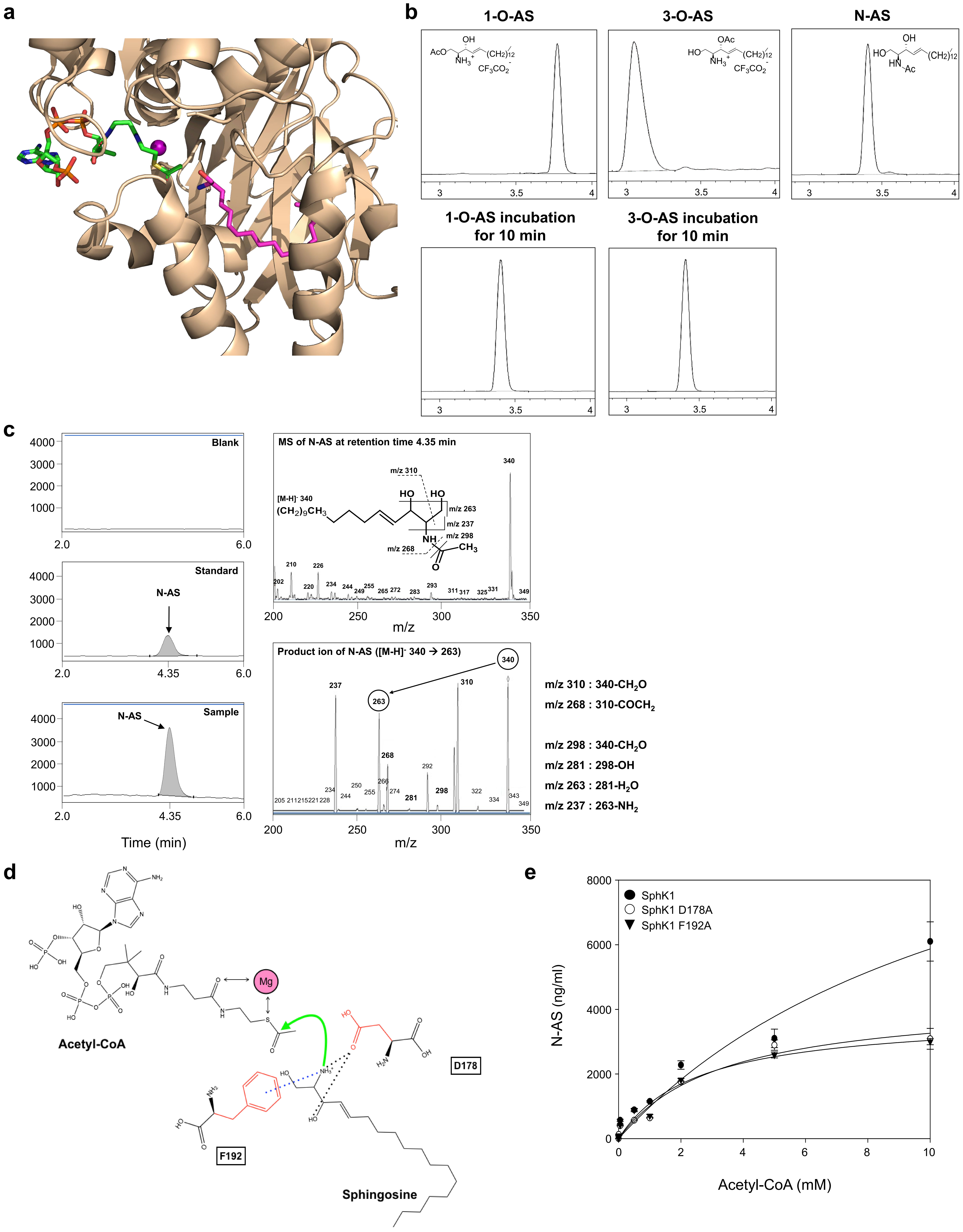

Supplementary Figure 2: SphK1 produces N-AS using sphingosine and acetyl-CoA. **a,** Ternary complex structure of acetyl-CoA and sphingosine docked into SphK1 based on the homology model (acetyl-CoA in green and sphingosine in pink). **b,** LC-ELSD analysis of synthesized compounds. Top left, 1-O-AS in ACN. Top middle, 3-O-AS in ACN. Top right, N-AS in ACN. Bottom left, 1-O-AS incubation for 10 min. Bottom right, 3-O-AS incubation for 10 min. **c,** Representative chromatograms of blank, N-AS standard, and N-AS in samples (left panel). Molecular MS scanning from the peak at retention time 4.35 min (right upper panel) and MS/MS fragmentation pattern of N-AS from the peak at retention time 4.35 min (right lower panel). **d,** Proposed mechanism involving acetylation of sphingosine. **e,** Quantification of N-AS in SphK1 WT, SphK1 D178A, and SphK1 F192A treated with 100 µM sphingosine and acetyl-CoA ranging from 0 - 10 mM (n = 3 independent experiments per group). All error bars indicate s.e.m.

a

	% of remaining after 0.5 h	Half life (h)
Human liver microsomes	50.1	0.6
Mouse liver microsomes	60.7	0.9

b

CYP inhibition							
CYP	3A4	2C9	2D6	1A2	2B6	2C19	2E1
IC_{50} (μM)	> 10	> 10	> 10	> 10	> 10	> 10	> 10
% of control at 25 μM	114.4	95.5	103.6	94.6	92.7	79.6	84.0

c

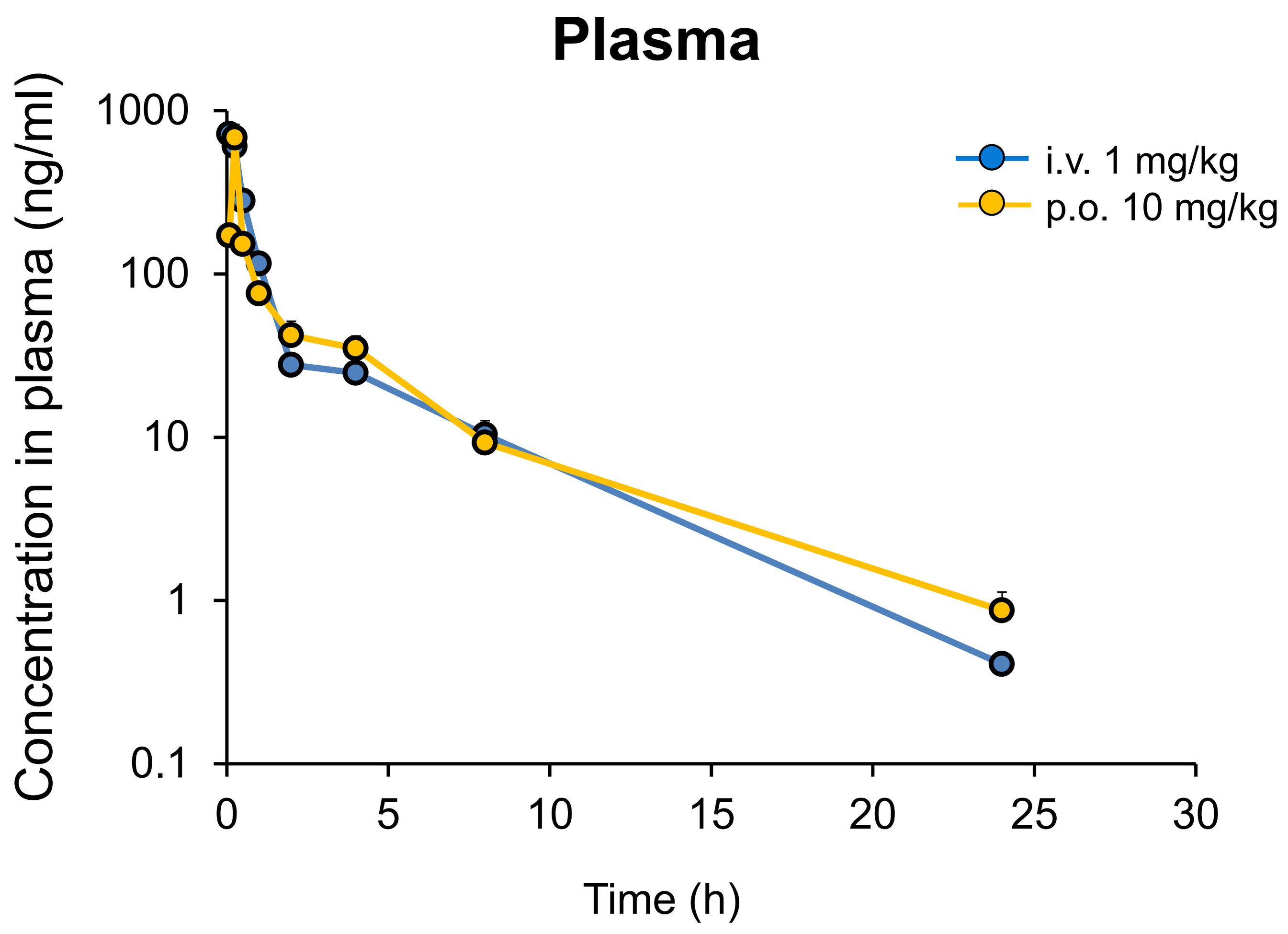

d

PK parameter (Plasma)	N-AS	
	p.o.10 mg/kg	i.v.1 mg/kg
AUC_{last} (ng·h/ml)	545.33±72.13	665.37±28.64
AUC_{inf} (ng·h/ml)	550.30±71.35	667.42±28.82
Half-life $_{plasma}$ (h)	3.68±0.14	3.45±0.16
MRT (h)	3.10±0.14	2.27±0.11
C_{max} (ng/ml)	682.75±141.13	/
Co (ng/ml)	/	854.36±128.60
T_{max} (h)	0.25±0.00	/
BA (%)	8.25	/

AUC, Area under curve to last time point; MRT, Mean resident time; BA, Bioavailability

e

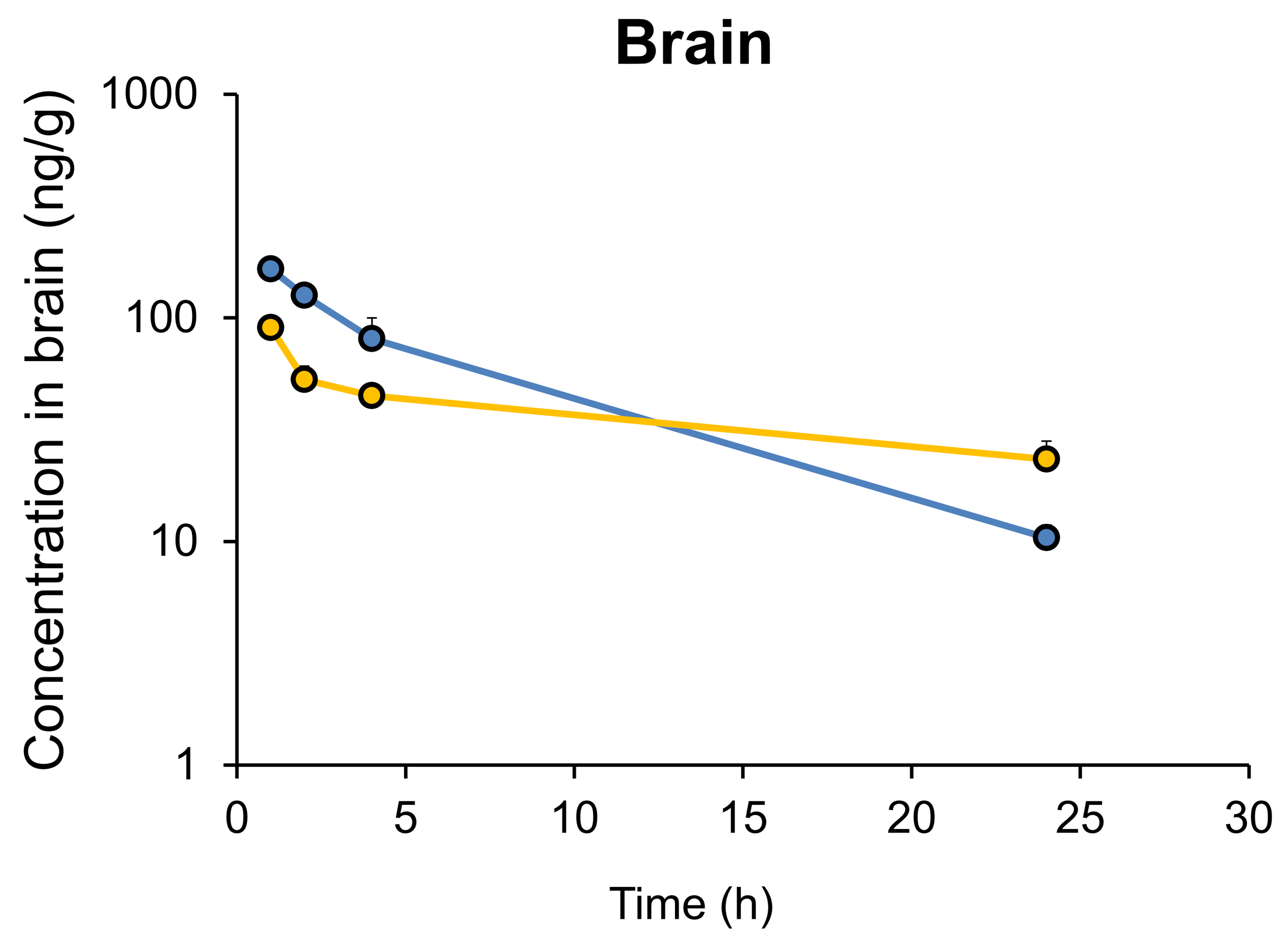

f

PK parameter (Brain)	N-AS	
	p.o.10 mg/kg	i.v.1 mg/kg
AUC_{last} (ng·h/ml)	895.32±60.53	1344.94±224.29
AUC_{inf} (ng·h/ml)	1748.43±361.71	1441.7±228.22
C_{max} (ng/ml)	90.27±2.95	165.18±18.42
Half-life $_{brain}$ (h)	21.99±6.07	6.31±0.38
MRT (h)	8.64±1.01	6.94±0.62
Brain distribution	3.18	2.16

AUC, Area under curve to last time point; MRT, Mean resident time

g

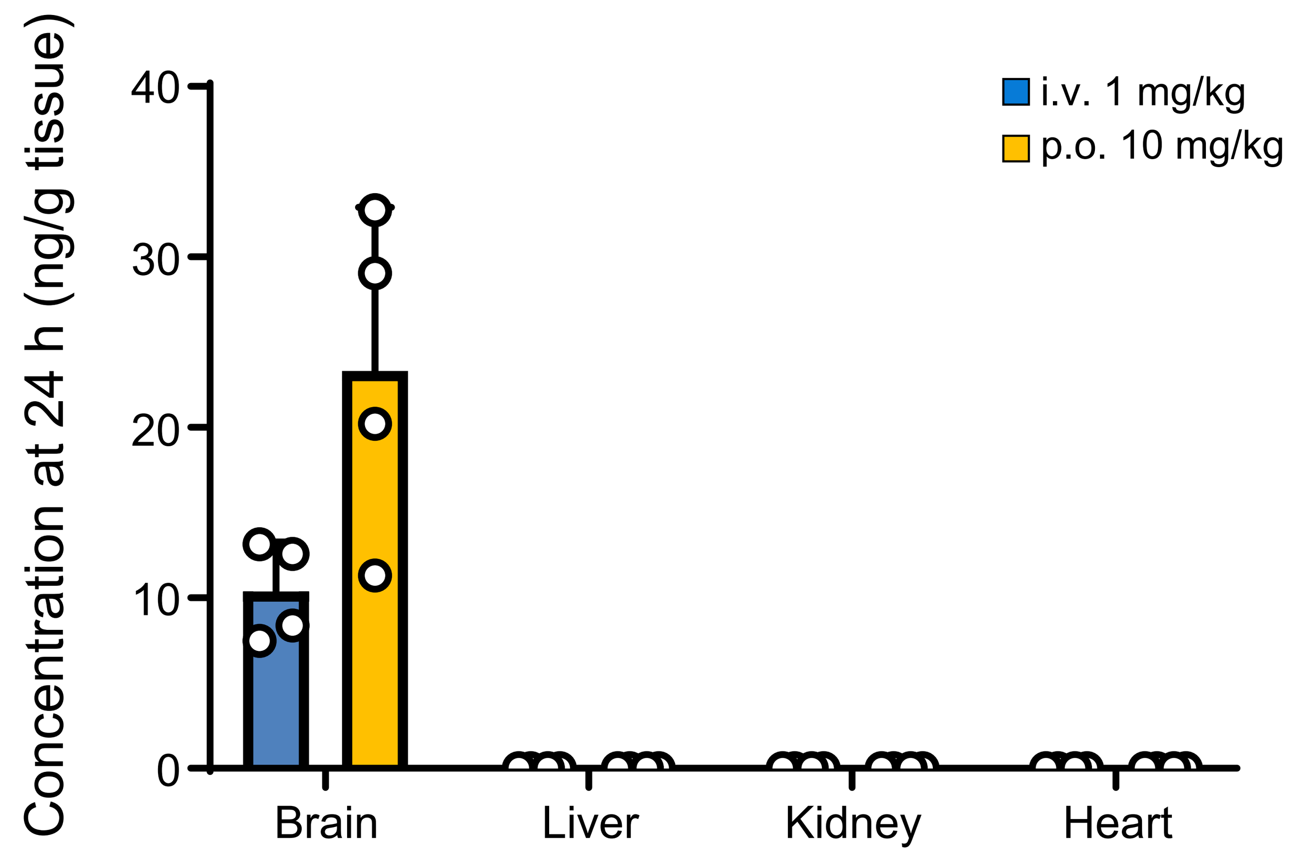

Supplementary Figure 3: PK studies of N-AS. **a,** % compound remaining after 0.5 h and the half-life (h) of N-AS after incubation with human and mouse liver microsomes (n = 3 independent experiments per group). **b,** The IC_{50} (μM) and % inhibition of 7 major CYP isozymes by N-AS at 25 μM (n = 3 independent experiments per group). **c-f,** Mean plasma (c) and brain (e) concentration vs. time profiles of N-AS after single dose i.v. and p.o. administration at doses of 1 mg kg^{-1} and 10 mg kg^{-1}, respectively (n = 4 mice per group). PK parameters of N-AS in plasma (d) and brain (f) after i.v. or p.o administration in mouse. **g,** Tissue concentrations of N-AS at 24 h in mouse following i.v. or p.o. delivery (n = 4 mice per group). All error bars indicate s.e.m. Source data are provided as a Source Data file.

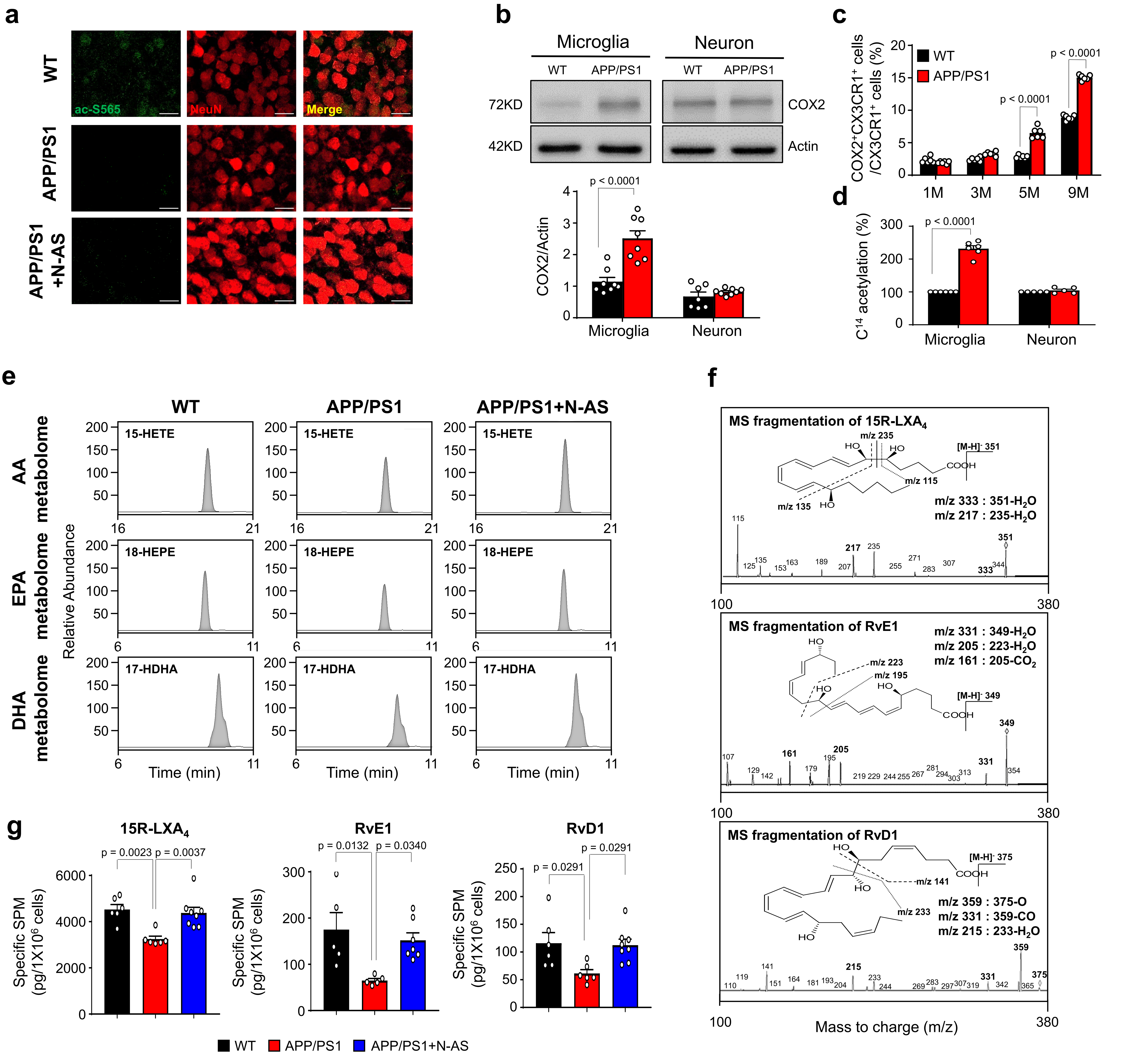

Supplementary Figure 4: Microglia are the likely target cells responsible for N-AS related AD pathogenesis and production of N-AS-triggered SPM. **a,** Immunofluorescence images of neuron (NeuN) with ac-S565 (green) in cortex of WT, APP/PS1, and APP/PS1 treated with N-AS mice brain. Scale bars, 50 µm. Data were replicated in three independent experiments with similar results. **b,** Western blotting for COX2 in microglia and neuron isolated from WT and APP/PS1 mouse cortex. (Microglia derived form APP/PS1 mice, n = 8; microglia derived from APP/PS1+N-AS mice, n = 8; neuron derived form APP/PS1 mice, n = 7; neuron derived from APP/PS1+N-AS mice, n = 8). **c,** Percentage of $COX2^{+}CX3CR1^{+}$ cells in $CX3CR1^{+}$ microglia cells from 1, 3, 5 or 9-mo-old WT and APP/PS1 mouse. (n = 6 mice per group). **d,** Acetylation assay of microglia and neuron isolated from WT and APP/PS1 mouse after oral administration of [^{14}C] N-AS. (n = 5-6 mice per group). **e,** Representative chromatogram showing the SPM precursors (Top, 15-HETE; Middle, 18-HEPE; Bottom, 17-HDHA) in microglia derived from WT, APP/PS1, and APP/PS1 injected with N-AS mice. **f,** Related MS/MS spectra employed for identification of LMs, based on analysis of microglia derived from APP/PS1 injected with N-AS mice. 15R-LXA_4, RvE1, and RvD1. **g,** Quantification of SPMs by LC-MS/MS in microglia derived from WT, APP/PS1, and APP/PS1 injected with N-AS mice (n = 5-8 mice per group). All data analysis was performed on 9-mo-old mice except c. b-d, Student's t test. g, One-way analysis of variance, Tukey's post hoc test. All error bars indicate s.e.m. Source data are provided as a Source Data file.

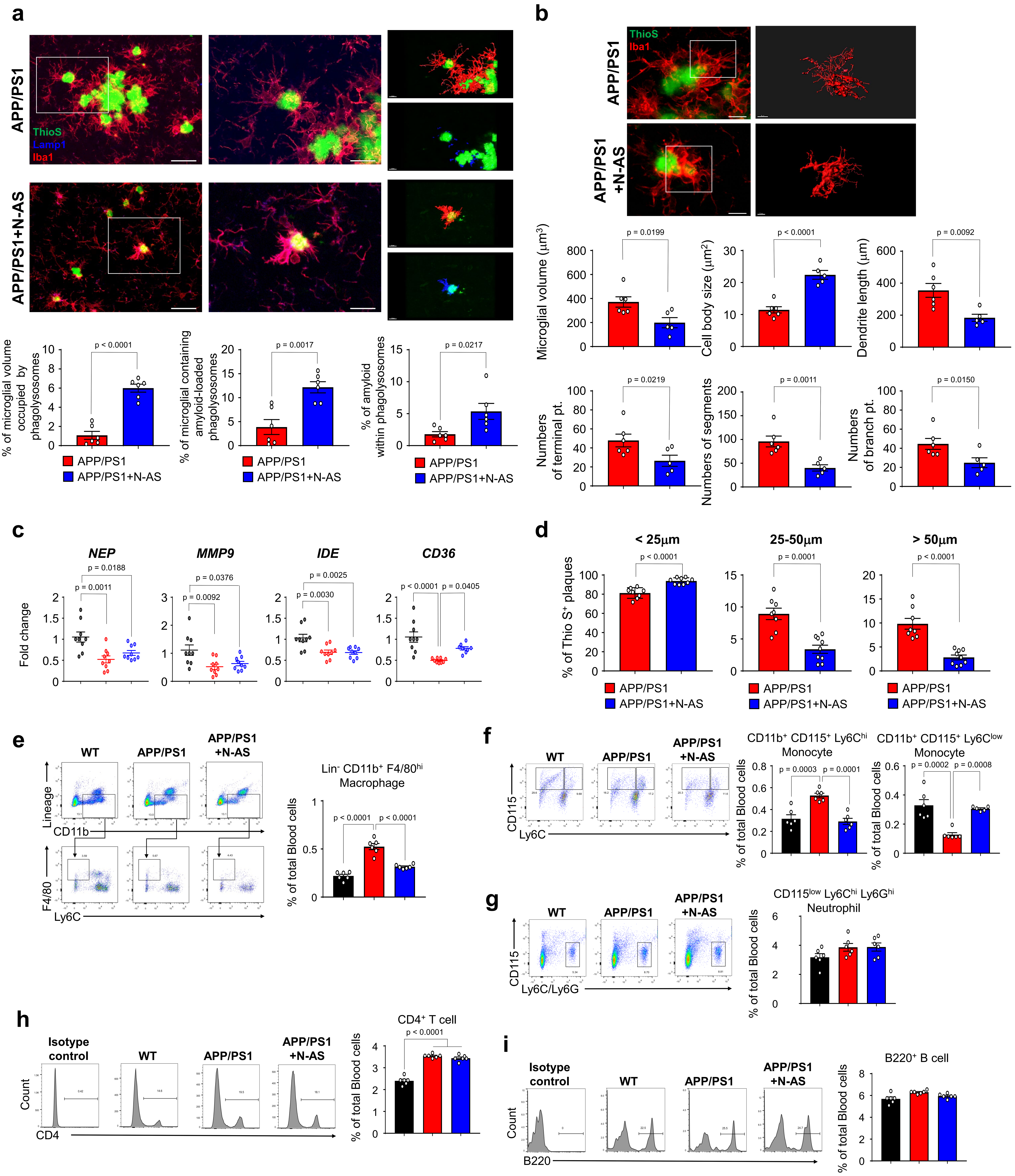

Supplementary Figure 5: N-AS administration ameliorates microglial phagocytosis and regulates peripheral immune cells in APP/PS1 mice. **a,** Top, immunofluorescence images of ThioS (Aβ plaques, green) encapsulated within Lamp1^{+} structures (phagolysosomes, blue) in microglia (Iba1, red) present in brains of APP/PS1 and APP/PS1 injected with N-AS mice. Low-magnification scale bars, 30 µm; High-magnification scale bars, 20 µm; 3D reconstruction from confocal image stacks scale bars, 10 µm. Bottom, quantification of microglia volume occupied by Lamp1^{+} phagolysosomes, percent of microglia containing Aβ-loaded phagolysosomes and Aβ encapsulated in phagolysosomes (n = 6 mice per group). **b,** Morphology of microglia (Iba1, red) surrounding Aβ (ThioS, green) in cortex of APP/PS1 and APP/PS1 injected with N-AS mice. Top, high magnification (Scale bars, 10 µm) and Imaris-based three-dimensional images (Scale bars, 5 µm) of microglia surrounding Aβ. Bottom, Imaris-based automated quantification of microglial morphology (APP/PS1 mice, n = 6; APP/PS1+N-AS mice, n = 5). **c,** mRNA levels of *NEP*, *MMP9*, *IDE*, and *CD36* expressions in microglia derived from WT, APP/PS1, and APP/PS1 injected with N-AS mice (n = 8-9 mice per group). **d** Morphometric analysis of Aβ plaques in APP/PS1 and APP/PS1 injected with N-AS mice (n = 8-9 mice per group). Brain sections were labeled with ThioS and plaques were counted and assigned to three mutually exclusive size categories based on maximum diameters: small <25 µm; medium 25-50 µm; or large >50 µm. **e,** Left, gating strategy for detection of Lin^{-}CD11b^{+}F4/80^{hi} macrophage within blood cell populations. Right, graph displaying the calculated percentage of Lin^{-}CD11b^{+}F4/80^{hi} macrophage in WT, APP/PS1, and APP/PS1 treated with N-AS mice blood (n = 6 mice per group). **f,** Left, gating strategy for detection of CD11b^{+}CD115^{+}Ly6C^{hi} (Pro-inflammatory monocyte) and CD11b^{+}CD115^{+}Ly6C^{low} (Anti-inflammatory monocyte) cells within blood cell populations. Right, graph displaying the calculated percentage of CD11b^{+}CD115^{+}Ly6C^{hi} (Pro-inflammatory monocyte) and CD11b^{+}CD115^{+}Ly6C^{low} (Anti-inflammatory monocyte) cells in WT, APP/PS1, and APP/PS1 treated with N-AS mice blood (n = 6 mice per group). **g,** Left, gating strategy for detection of CD115^{low}Ly6C^{hi}Ly6G^{hi} neutrophil within blood cell populations. Right, graph displaying the calculated percentage of CD115^{low}Ly6C^{hi}Ly6G^{hi} neutrophil in WT, APP/PS1, and APP/PS1 treated with N-AS mice blood (n = 6 mice per group). **h,** Left, histograms are representative of CD4^{+} T cell proliferation at blood. Right, graph displaying the calculated percentage of CD4^{+} T cell in WT, APP/PS1, and APP/PS1 treated with N-AS mice blood (n = 6 mice per group).**i,** Left, histograms are representative of B220^{+} B cell proliferation at blood. Right, graph displaying the calculated percentage of B220^{+} B cell in WT, APP/PS1, and APP/PS1 treated with N-AS mice blood (n = 6 mice per group). a-b and d, Student's t test. c and e-i, One-way analysis of variance, Tukey's post hoc test. All error bars indicate s.e.m. Source data are provided as a Source Data file.

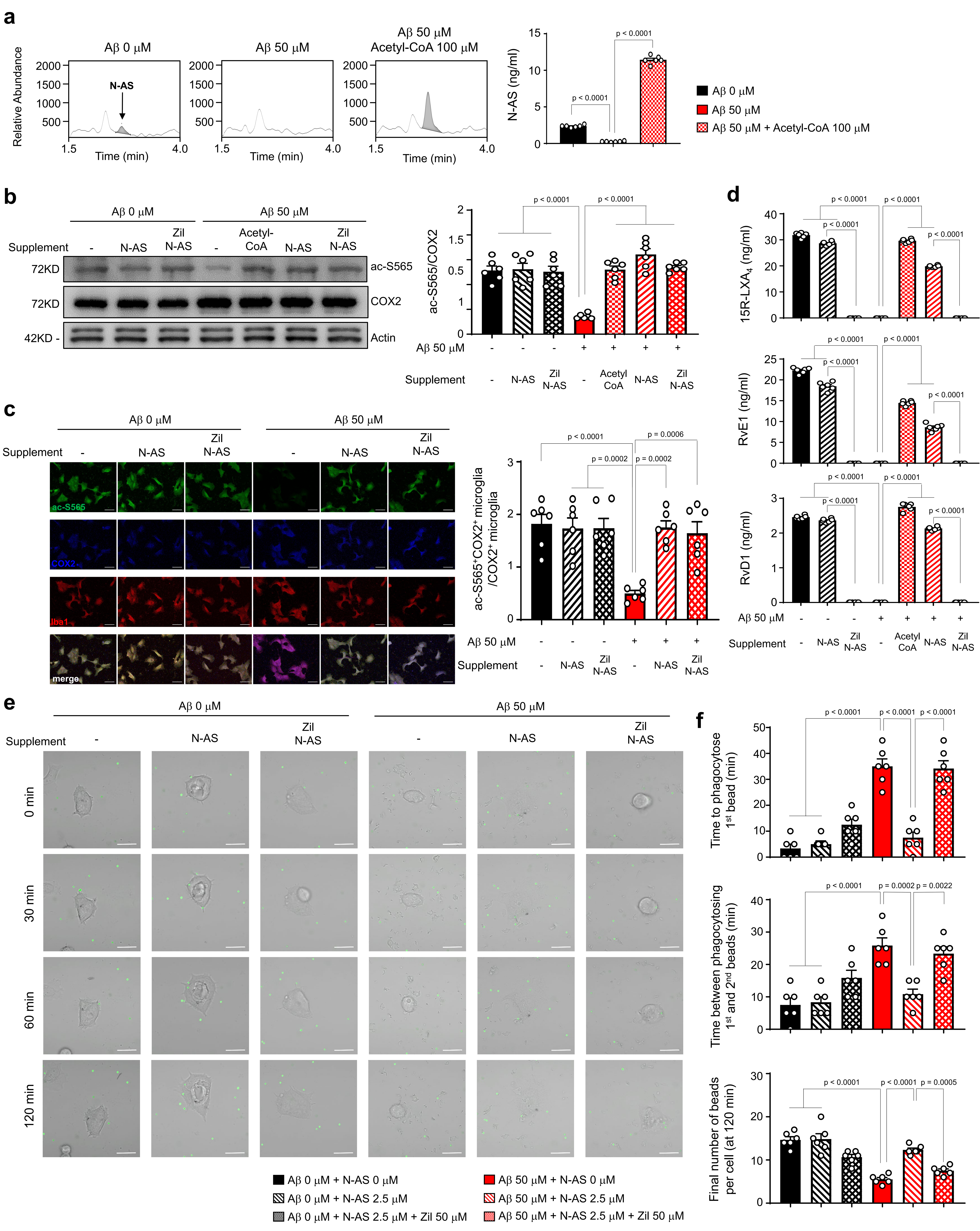

Supplementary Figure 6: Generation and function of N-AS is decreased in Aβ-treated human microglia, is restored with N-AS treatment, increasing phagocytic microglial function. **a,** Representative chromatograms of N-AS and quantification using systematic LC-MS/MS in human microglia treated 50 μM Aβ or not in presence or absence of acetyl-CoA (n = 6 per group). **b,** Western blot analysis for ac-S565 and total COX2 in human microglia treated 50 μM Aβ or not in presence or absence of N-AS or acetyl-CoA or zileuton (zil) (n = 6 per group). **c,** Colocalization of microglia (Iba1, red) with ac-S565 (green) and COX2 (blue) and quantification in human microglia treated 50 μM Aβ or not in presence or absence of N-AS or zil (n = 6 per group, Scale bars, 50 μm). **d,** Quantifications of 15R-LXA$_4$, RvE1, and RvD1 were detected by LC-MS/MS in cell lysates of human microglia treated with or without 50 μM Aβ in presence or absence of N-AS, acetyl-CoA, or zil (n = 6 per group). **e,** Representative images from live-cell imaging at various times after the administration of beads in human microglia treated 50 μM Aβ or not in presence or absence of N-AS or zil. Scare bars, 30 μm. Data were replicated in six independent experiments with similar results. **f,** Analysis of beads uptake in human microglia treated 50 μM Aβ or not in presence or absence of N-AS or zil. The amount of time taken to phagocytose the first beads (top), the time between phagocytosing the first and second beads (middle) and the final number of beads phagocytosed in 120 min (bottom) (n = 6 per group). a-d, and f, One-way analysis of variance, Tukey's post hoc test. All error bars indicate s.e.m. Source data are provided as a Source Data file.

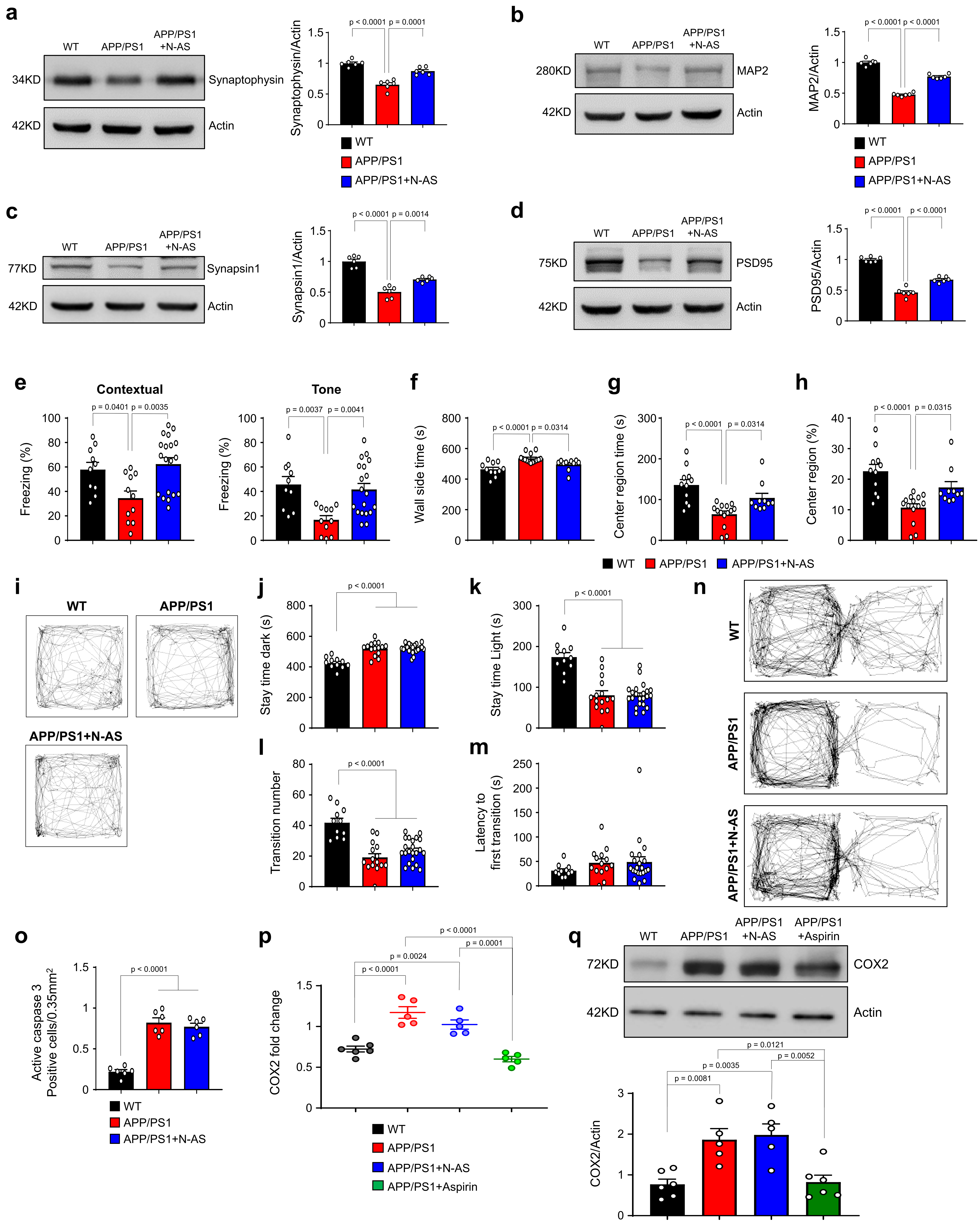

Supplementary Figure 7: N-AS administration restores synaptogenesis and AD-like behavior in APP/PS1 by N-AS-triggered SPMs, not apoptosis and COX2 inhibition. **a-d,** Western blot analysis for synaptophysin (a; n = 6 per group), MAP2 (b; n = 6 per group), synapsin1 (c; n = 6 per group) and PSD95 (d; n = 6 per group) in cortex of WT, APP/PS1, and APP/PS1 treated with N-AS. **e,** The results of contextual and tone tasks during fear conditioning test in WT, APP/PS1, and APP/PS1 treated with N-AS mice (n = 10-19 mice per group). **f and g,** Time spent in the walls side (f) and center regions (g) across during open field test of WT, APP/PS1, and APP/PS1 treated with N-AS mice (n = 10-14 mice per group). **h,** Percent of center region during open field test. **i,** Representative traces of mouse movement during open field test. **j and k,** Time spent in the dark (j) and light (k) compartment during light-dark test of WT, APP/PS1, and APP/PS1 treated with N-AS mice (n = 11-22 mice per group). **l**, The number of light-dark transition during light-dark test. **m,** Latency to the first transition during light-dark test. **n,** Representative traces of mouse movement during light-dark test. **o,** Quantification of Caspase-3$^+$ cells in WT, APP/PS1, and APP/PS1 treated with N-AS (n = 6 per group). **p,** mRNA level of *COX2* in microglia derived from WT, APP/PS1, APP/PS1 treated with N-AS and APP/PS1 treated with aspirin mice (n = 5-6 mice per group). **q,** Western blot analysis for COX2 in microglia derived from WT, APP/PS1, APP/PS1 treated with N-AS, and APP/PS1 treated with aspirin mice (WT mice, n = 6; APP/PS1 mice, n = 5; APP/PS1+N-AS mice, n = 5; APP/PS1+Aspirin mice, n = 6). All data analysis was done at 9-mo-old mice. a-h, j-m, and o-q, One-way analysis of variance, Tukey's post hoc test. All error bars indicate s.e.m. Source data are provided as a Source Data file. Source data are provided as a Source Data file.

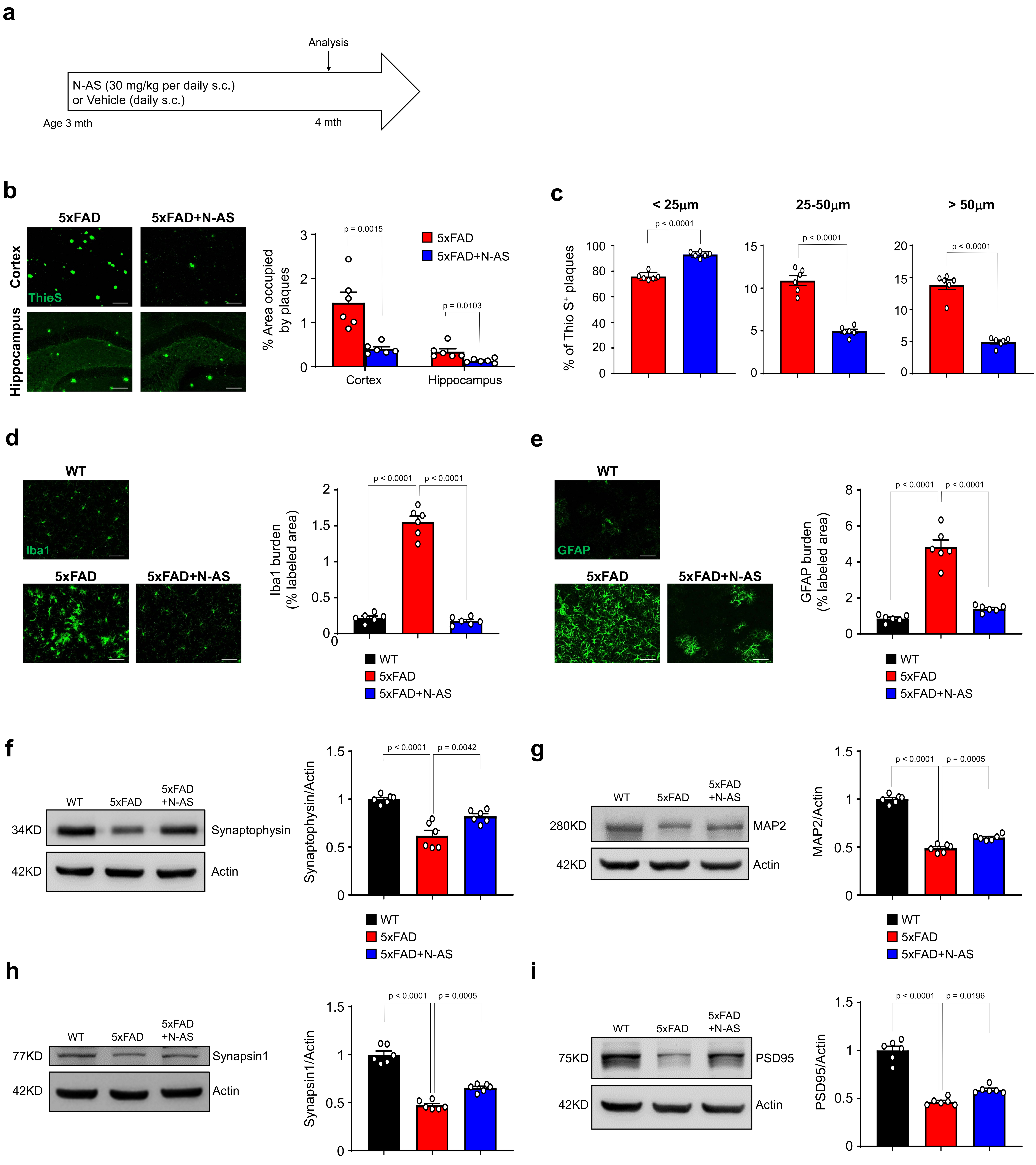

Supplementary Figure 8: N-AS treatment reduces AD pathology in 5xFAD mice. **a,** Scheme of experimental procedure. Daily administration of N-AS or vehicle subcutaneously to 3-mo-old 5xFAD mice for 4 weeks. **b,** Left, representative immunofluorescence images of thioflavin S (ThoS, Aβ plaques) in cortex (Scale bars, 50 µm) and hippocampus (Scale bars, 100 µm) of 5xFAD and 5xFAD treated with N-AS mice. Right quantification of area occupied by Aβ plaques (n = 6 mice per group). **c,** Morphometric analysis of Aβ plaques in 5xFAD and 5xFAD injected with N-AS mice (n = 6 mice per group). Brain sections were labeled with ThioS and plaques were counted and assigned to three mutually exclusive size categories based on maximum diameters: small <25 µm; medium 25-50 µm; or large >50 µm. **d,** Left, immunofluorescence images of microglia (Iba1) in cortex of WT, 5xFAD, and 5xFAD treated with N-AS mice brain. Scale bars, 100 µm. Right, quantification of $Iba1^+$ microglia in WT, 5xFAD, and 5xFAD treated with N-AS (n = 6 mice per group). **e,** Left, immunofluorescence images of astrocyte (GFAP) in cortex of WT, 5xFAD, and 5xFAD treated with N-AS mice brain. Scale bars, 100 µm. Right, quantification of $GFAP^+$ astrocyte in WT, 5xFAD, and 5xFAD treated with N-AS (n = 6 mice per group). **f-I,** Western blot analysis for synaptophysin (f; n = 6 per group), MAP2 (g; n = 6 per group), synapsin1 (h; n = 6 per group), and PSD95 (i; n = 6 per group) in cortex of WT, 5xFAD, and 5xFAD treated with N-AS. All data analysis was done at 4-mo-old mice. b and c, Student's t test. d-i, One-way analysis of variance, Tukey's post hoc test. All error bars indicate s.e.m. Source data are provided as a Source Data file.

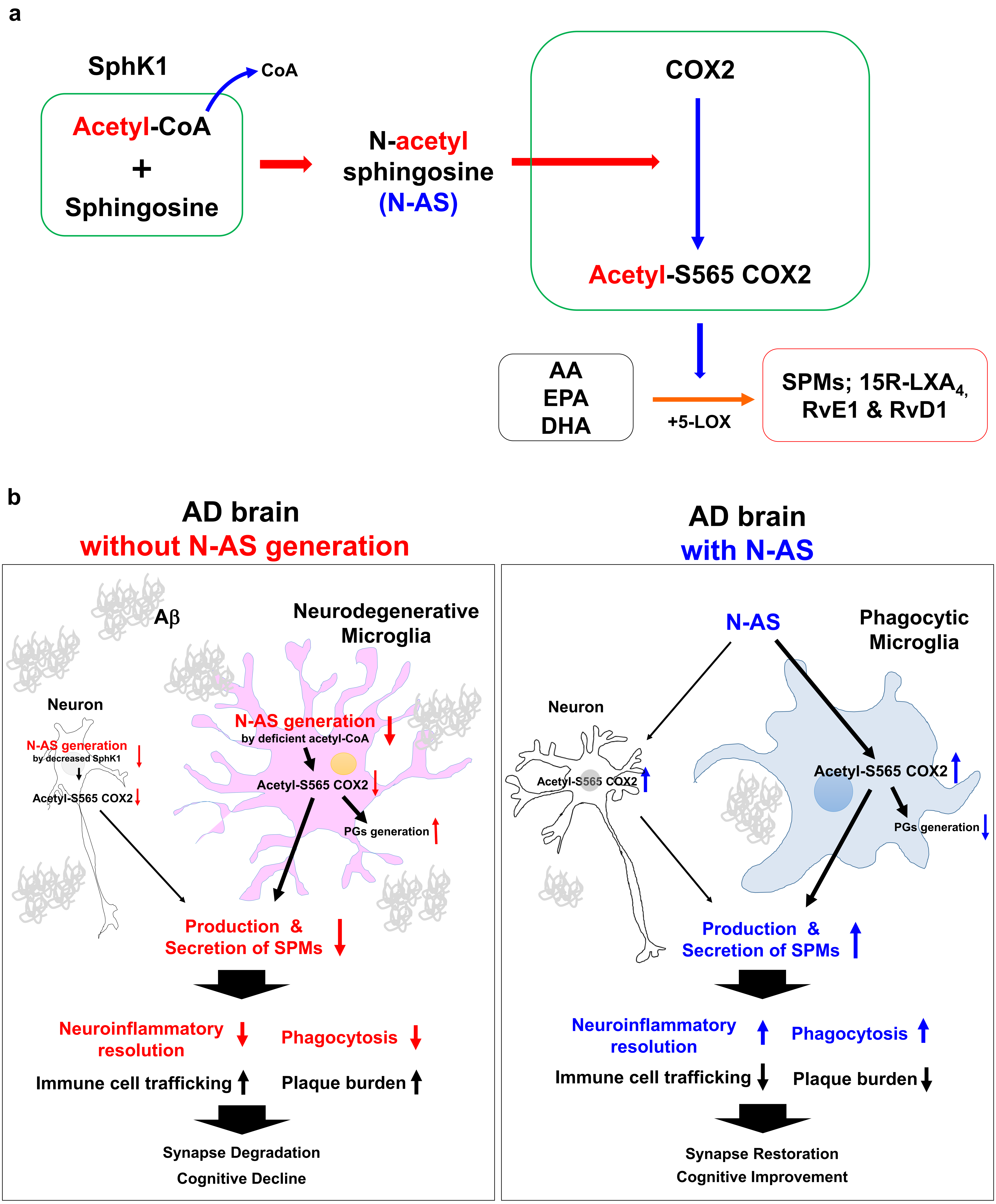

Supplementary Figure 9: Biosynthetic mechanism and functions of N-AS. **a,** N-AS is synthesized by acetyl-CoA and sphingosine within SphK1. The N-AS acetylates S565 in COX2, and N-AS-acetylated COX2 generates SPMs in the presence of 5-LOX. **b,** In AD brain, N-AS generation in neuron and neurodegenerative microglia was decreased by deficiency of neuronal SphK1 and microglial acetyl-CoA, followed reduction of production and secretion of SPMs through decreased acetyl-S565 COX2. The decreased N-AS-triggered SPMs caused failure of neuroinflammatory resolution and reduced phagocytosis ability, leading to increased immune cell trafficking and plaque burden, exacerbating AD pathology (Left). When N-AS was treated to AD mice, COX2 was acetylated in residue S565 by N-AS, and it increases production and secretion of SPMs, promoting resolution of inflammation and phagocytosis. This induced decrease of immune cell trafficking and plaque burden, resulting in ameliorating AD pathology (Right).

ac-S565 / COX2 in recombinant COX2 (Fig. 1e)

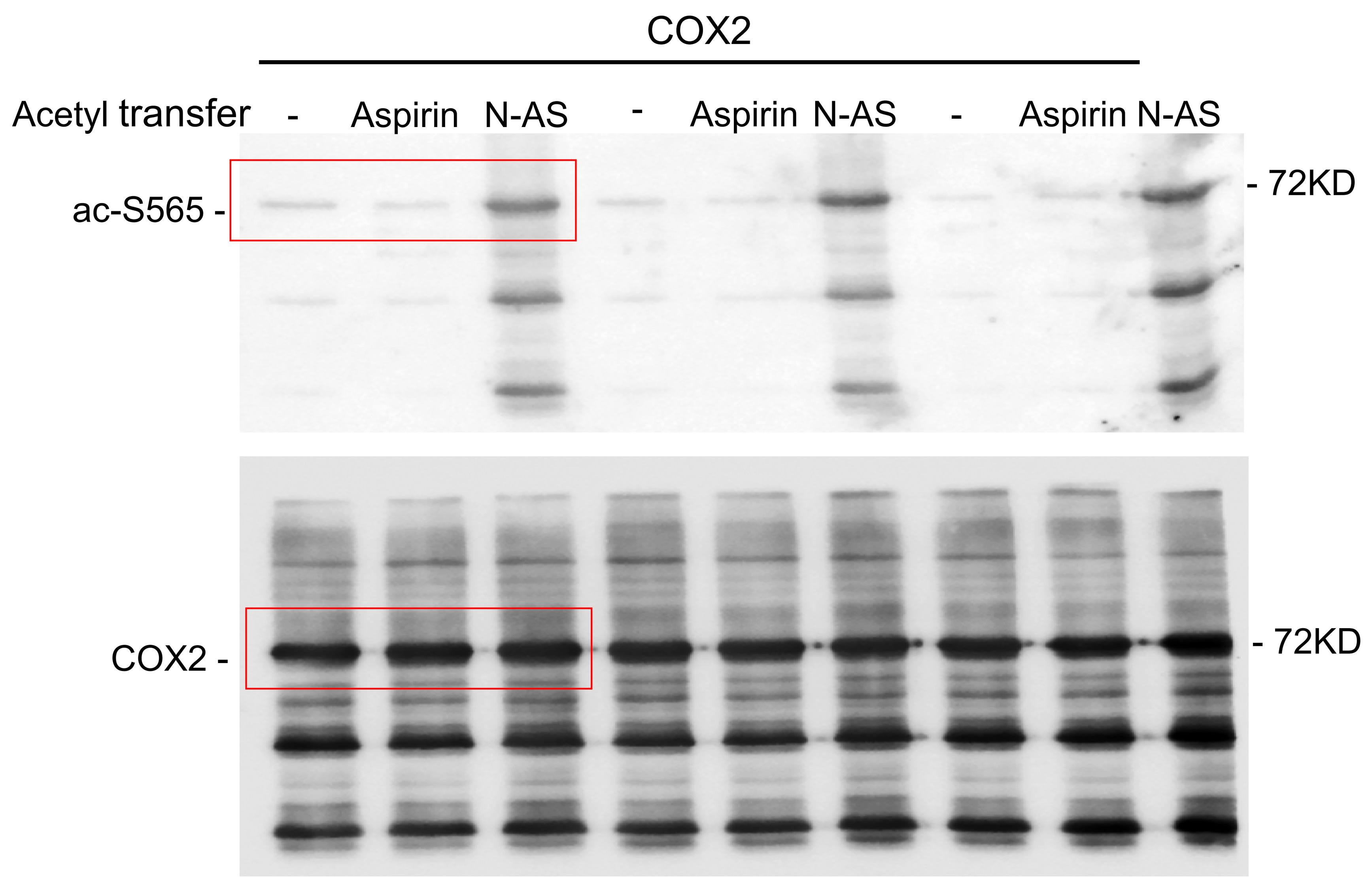

ac-S565 / COX2 in neuron, microglia, astrocyte (Fig. 3b)

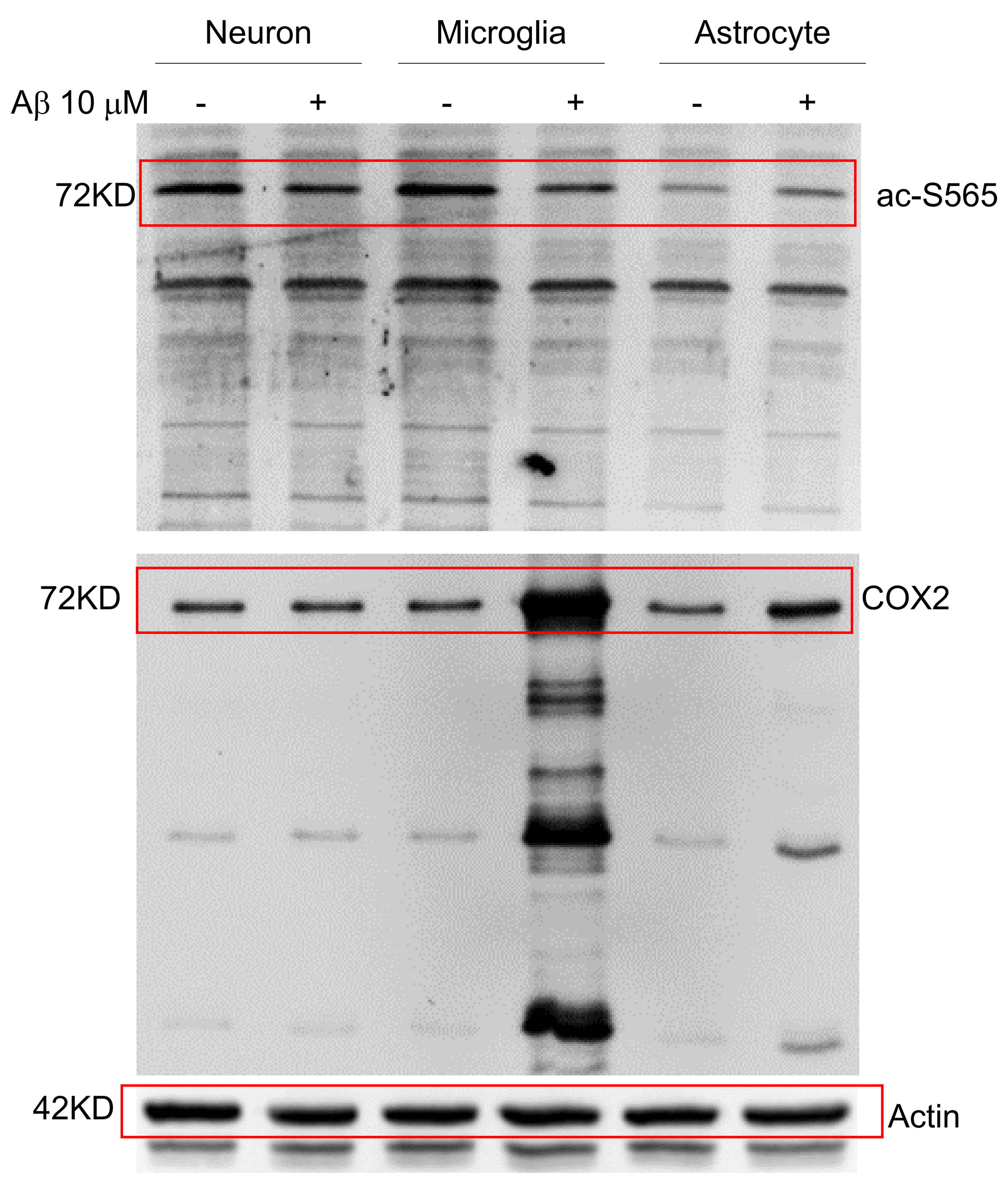

ac-S565 / COX2 in neuron (Fig. 3h)

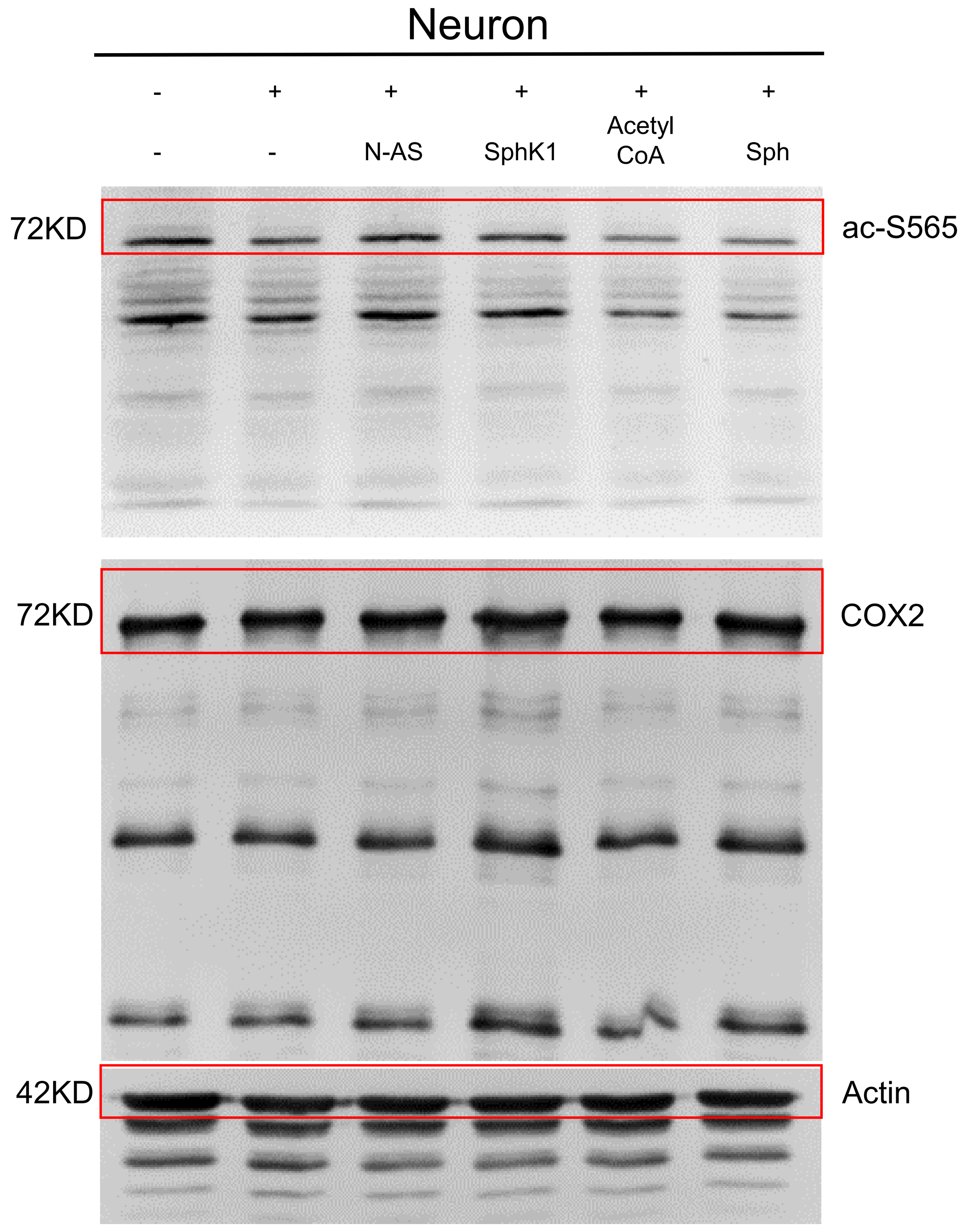

ac-S565 / COX2 in microglia(Fig. 3h)

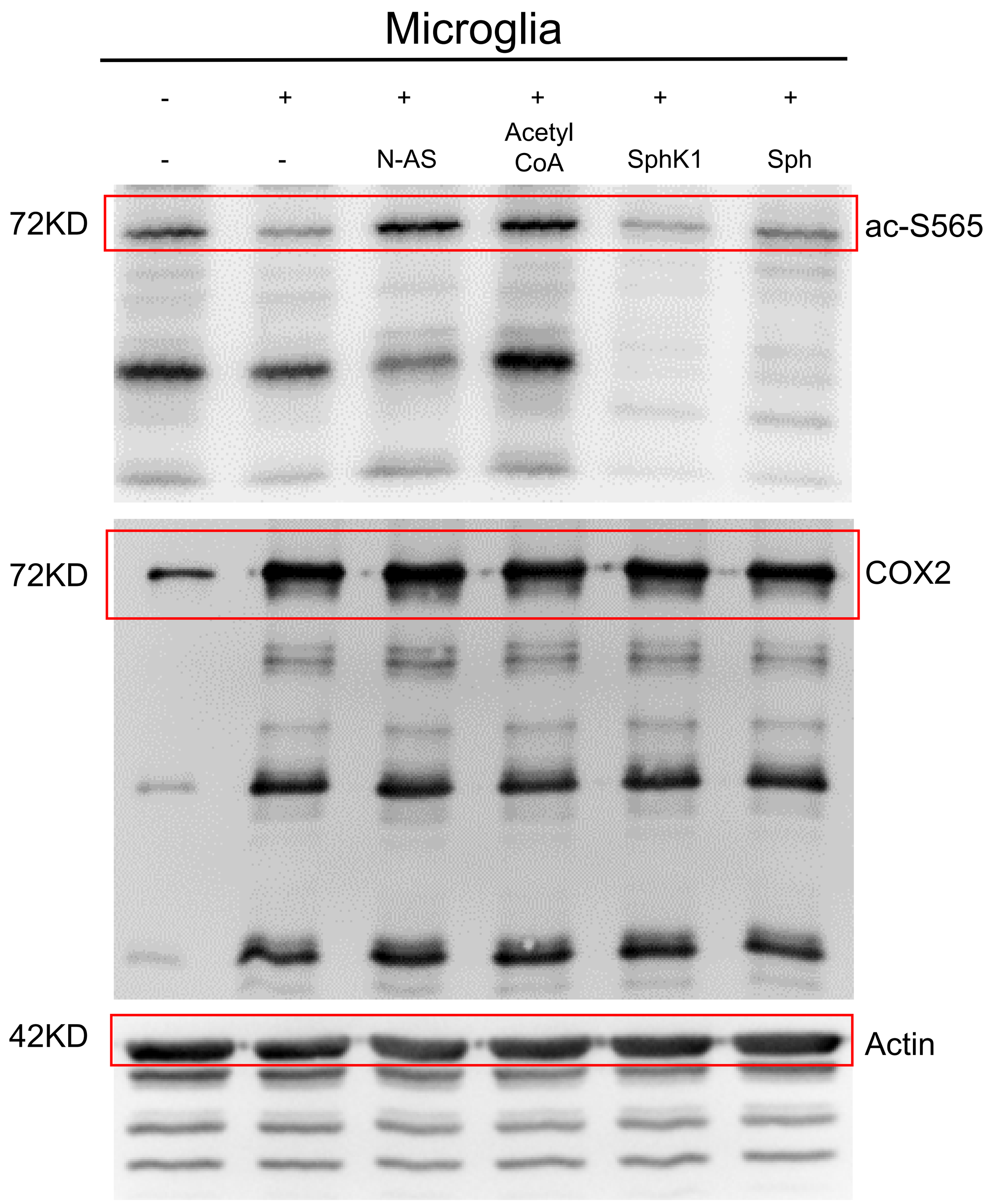

Supplementary Figure 10: Full scans of the western blots. Boxes indicate cropped images used in the figures and numbers indicate the molecular weight.

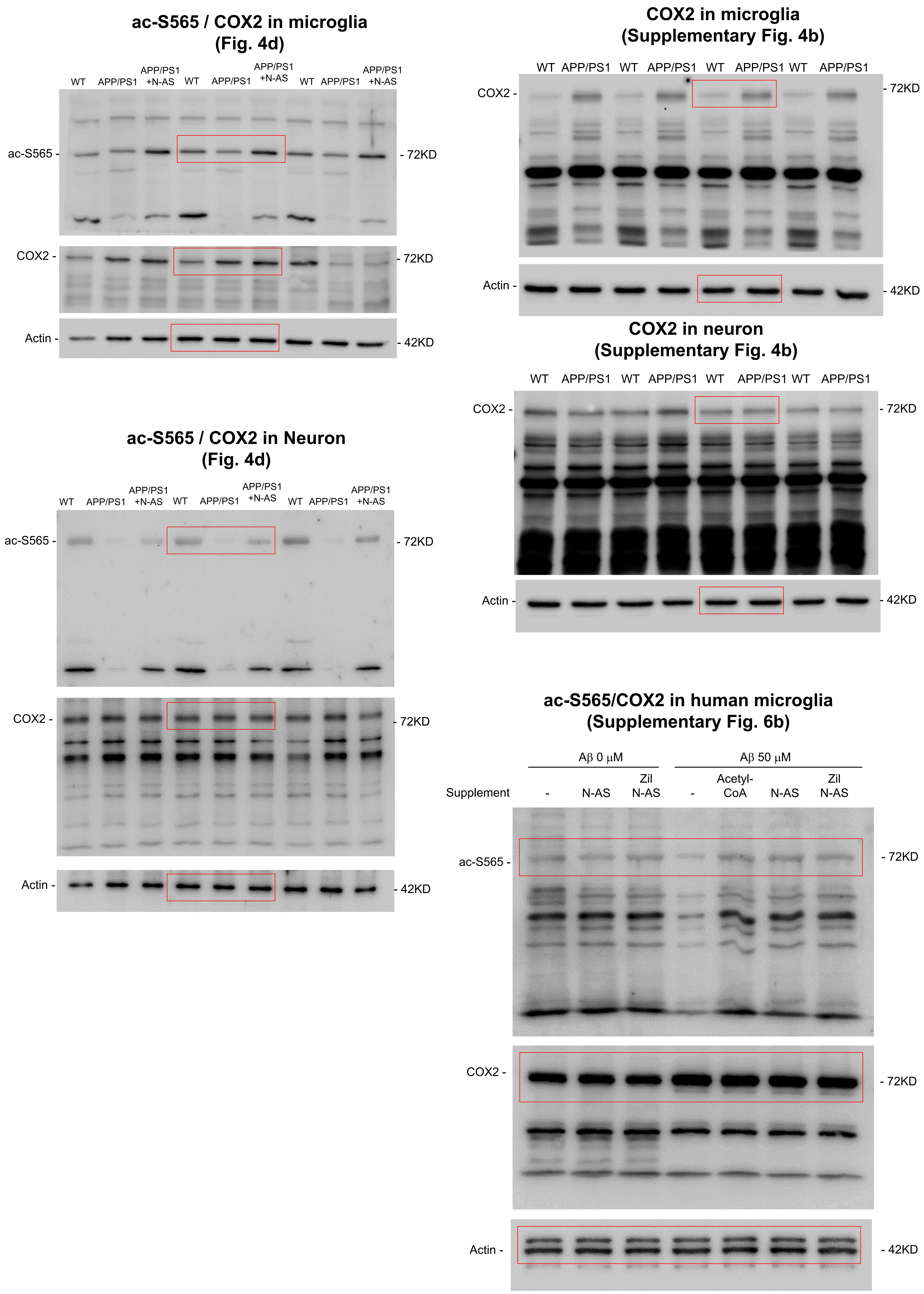

Supplementary Figure 10: Full scans of the western blots. Boxes indicate cropped images used in the figures and numbers indicate the molecular weight.

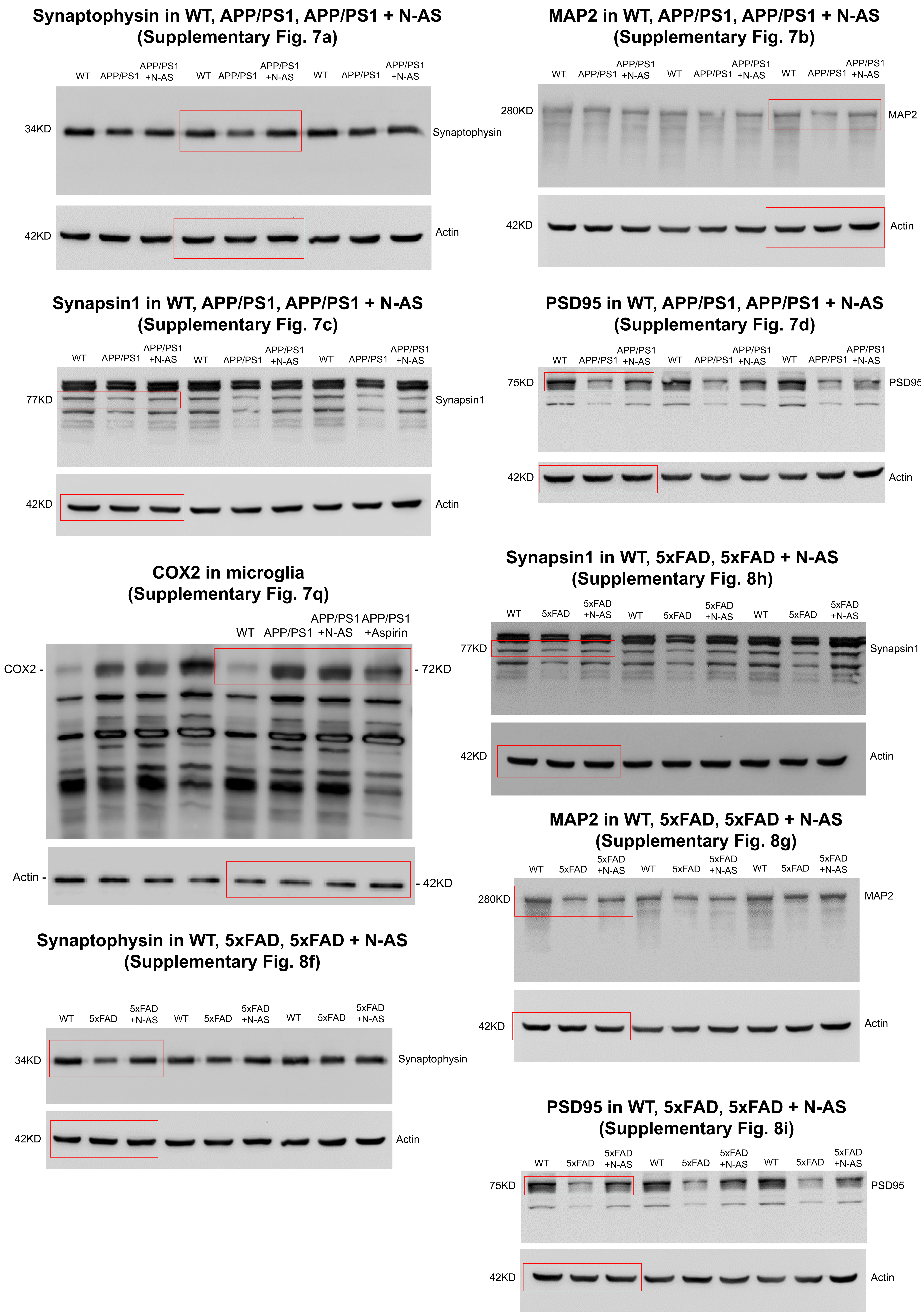

Supplementary Figure 10: Full scans of the western blots. Boxes indicate cropped images used in the figures and numbers indicate the molecular weight.
