# Supplementary material for: N-AS-triggered SPMs are direct regulators of microglia in a model of Alzheimer’s disease
Source: Nat Commun. 2020 May 12;11:2358. doi: 10.1038/s41467-020-16080-4 (PMC7217877; doi:10.1038/s41467-020-16080-4)
Supplement: Supplementary file 3 — Description of Additional Supplementary Information [file 41467_2020_16080_MOESM3_ESM.pdf]

Description of Additional Supplementary Files

File Name: Supplementary Data 1
Description: Synthesis of acetyl sphingosines

File Name: Supplementary Data 2
Description: H and 13 1 C NMR Spectra.

File Name: Supplementary Movie 1
Description: Phagocytosis of human microglia treated without Aβ and N-AS.
Human microglia treated without N-AS phagocytoses FITC-beads in absence of Aβ (Supplementary Fig. 6e, representative image from 6 experiments). Images were acquired at 5 min intervals. Scale bars, 30 μm.

File Name: Supplementary Movie 2
Description: Phagocytosis of human microglia treated N-AS in absence of Aβ
Human microglia treated with N-AS phagocytoses FITC-beads in absence of Aβ (Supplementary Fig. 6e, representative image from 6 experiments). Images were acquired at 5 min intervals. Scale bars, 30 μm.

File Name: Supplementary Movie 3
Description: Phagocytosis of human microglia treated N-AS and zil in absence of Aβ
Human microglia treated with N-AS and zil phagocytoses FITC-beads in absence of Aβ (Supplementary Fig. 6e, representative image from 6 experiments). Images were acquired at 5 min intervals. Scale bars, 30 μm.

File Name: Supplementary Movie 4
Description: Phagocytosis of human microglia treated without N-AS in presence of Aβ
Human microglia treated without N-AS phagocytoses FITC-beads in presence of Aβ (Supplementary Fig. 6e, representative image from 6 experiments). Images were acquired at 5 min intervals. Scale bars, 30 μm.

File Name: Supplementary Movie 5
Description: Phagocytosis of human microglia treated with N-AS in presence of Aβ
Human microglia treated with N-AS phagocytoses FITC-beads in presence of Aβ (Supplementary Fig. 6e, representative image from 6 experiments). Images were acquired at 5 min intervals. Scale bars, 30 μm.

File Name: Supplementary Movie 6
Description: Phagocytosis of human microglia treated with N-AS and zil in presence of Aβ
Human microglia treated with N-AS and zil phagocytoses FITC-beads in presence of Aβ (Supplementary Fig. 6e, representative image from 6 experiments). Images were acquired at 5 min intervals. Scale bars, 30 μm.
