# Supplementary figures and images for: N-AS-triggered SPMs are direct regulators of microglia in a model of Alzheimer’s disease
Source: Nat Commun. 2020 May 12;11:2358. doi: 10.1038/s41467-020-16080-4 (PMC7217877; doi:10.1038/s41467-020-16080-4)

**Supplementary Data 1.** Synthesis of acetyl sphingosines

**
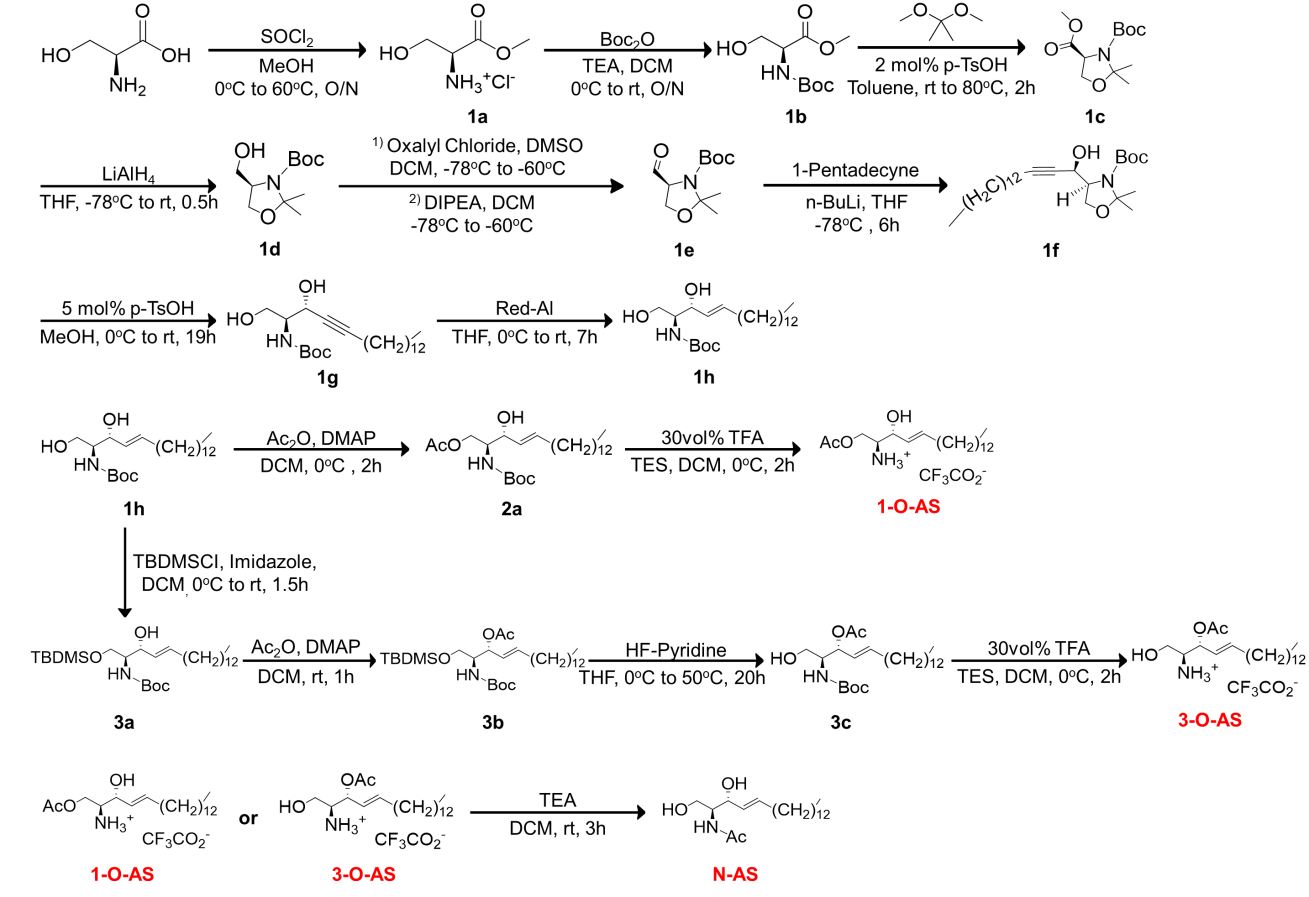
**

Supplement: Supplementary file 4 — Supplementary Data 1 [file 41467_2020_16080_MOESM4_ESM.docx]
