# Supplementary material for: N-AS-triggered SPMs are direct regulators of microglia in a model of Alzheimer’s disease
Source: Nat Commun. 2020 May 12;11:2358. doi: 10.1038/s41467-020-16080-4 (PMC7217877; doi:10.1038/s41467-020-16080-4)
Supplement: Supplementary file 5 — Supplementary Data 2 [file 41467_2020_16080_MOESM5_ESM.docx]

**Supplementary Data 2.** ^1^H and ^13^C NMR Spectra.

**1a** ^1^H-NMR

**1a** ^13^C-NMR

**1b** ^1^H-NMR

**1b** ^13^C-NMR

**1c** ^1^H-NMR

**1c** ^13^C-NMR

**1d** ^1^H-NMR

**1d** ^13^C-NMR

**1f** ^1^H-NMR

**1f** ^13^C-NMR

**1g** ^1^H-NMR

**1g** ^13^C-NMR

**1h** ^1^H-NMR

**1h** ^13^C-NMR

**2a** ^1^H-NMR

**2a** ^13^C-NMR

**1-O-AS** ^1^H-NMR

**1-O-AS** ^13^C-NMR

**3a** ^1^H-NMR

**3a** ^13^C-NMR

**3b** ^1^H-NMR

**3b** ^13^C-NMR

**3c** ^1^H-NMR

**3c** ^13^C-NMR

**3-O-AS** ^1^H-NMR

**3-O-AS** ^13^C-NMR

**N-AS** ^1^H-NMR

**N-AS** ^13^C-NMR
