# Supplementary material for: N-AS-triggered SPMs are direct regulators of microglia in a model of Alzheimer’s disease
Source: Nat Commun. 2020 May 12;11:2358. doi: 10.1038/s41467-020-16080-4 (PMC7217877; doi:10.1038/s41467-020-16080-4)
Supplement: Supplementary file 12 — Reporting Summary [file 41467_2020_16080_MOESM12_ESM.pdf]

nature research

Corresponding author(s): Jae-sung Bae

Last updated by author(s): Feb 18, 2020

Reporting Summary

Nature Research wishes to improve the reproducibility of the work that we publish. This form provides structure for consistency and transparency in reporting. For further information on Nature Research policies, see Authors & Referees and the Editorial Policy Checklist .

Statistics

For all statistical analyses, confirm that the following items are present in the figure legend, table legend, main text, or Methods section.

n/a	Confirmed	
☐	☒	The exact sample size (*n*) for each experimental group/condition, given as a discrete number and unit of measurement
☐	☒	A statement on whether measurements were taken from distinct samples or whether the same sample was measured repeatedly
☐	☒	The statistical test(s) used AND whether they are one- or two-sided<br>*Only common tests should be described solely by name; describe more complex techniques in the Methods section.*
☒	☐	A description of all covariates tested
☐	☒	A description of any assumptions or corrections, such as tests of normality and adjustment for multiple comparisons
☐	☒	A full description of the statistical parameters including central tendency (e.g. means) or other basic estimates (e.g. regression coefficient) AND variation (e.g. standard deviation) or associated estimates of uncertainty (e.g. confidence intervals)
☐	☒	For null hypothesis testing, the test statistic (e.g. *F*, *t*, *r*) with confidence intervals, effect sizes, degrees of freedom and *P* value noted<br>*Give P values as exact values whenever suitable.*
☒	☐	For Bayesian analysis, information on the choice of priors and Markov chain Monte Carlo settings
☒	☐	For hierarchical and complex designs, identification of the appropriate level for tests and full reporting of outcomes
☐	☒	Estimates of effect sizes (e.g. Cohen's *d*, Pearson's *r*), indicating how they were calculated

Our web collection on statistics for biologists contains articles on many of the points above.

Software and code

Policy information about availability of computer code

Data collection	The numbers of samples used to result in statistically significant differences was calculated using G-power software with a significance level of 0.05 and a power of 0.8.
Data analysis	All software used for analysis is commercially available. Molecular modeling studies were performed using Discovery Studio Programs (2018, Accelrys) and figures were generated using PyMOL programs. For Sphk1 activity, S1P and sphingosine quantification, Waters Millennium software was used. For kinetic analysis, Sigma-plot program (ver 10.0, Systat software Inc.) was used. To identify acetylation site of COX2 using LC-MS/MS system, BioTools 3.2 SR5 (Bruker Daltonics) was used. The peak areas of lipid mediators were automatically integrated using Mass Hunter B 06.00. The PK parameters were evaluated using Pheonix WinNonlin 5.1 (Princeton). For histologcal analysis, MetaMorph software (Molecular Devices) or IMARIS software (ver 8, Bitplane) were used. For the analysis of microglial COX2+ CX3CR1+ cells, the images were acquired on Alexa 488 and APC Channel with performed using Operetta CLS High-Content Analysis System (Perkin-Elmer) and Analysis Software 4.5 (Perkin-Elmer). For densitometric quantification of western blot, ImageJ software (National Institutes of Health) was used. In flow cytometry, fluorescence data were acquired on a BD FACSAriaIII flow cytometer (BD) using Diva software (BD) and further analyzed using FlowJo analytical software (ver 10, Tree Star, Inc.). For live-cell phagocytosis assay, a laser scanning confocal microscope equipped with live cell chamber system (FV3000, Olympus) was used for 2 h. Confocal images were collected every 5 min for 2 h and analyzed using the ImageJ software (National Institutes of Health). All statistical data were graphed and comparisons between two groups were performed with Student's t-test. In cases where more than two groups were compared to each other, a one way analysis of variance (ANOVA) was used, followed by Tukey's HSD test. All statistical analysis was performed using SPSS statistical software (IBM, ver 23.0) and GraphPad Prism 7.0 software. This programs provide whether our data come from normal distribution. We checked the normal distribution using Shaprio-Wilk analysis. When the data existed in normal distribution, we progress further analysis. This programs also provides the equality of variances. For RNA seq functional gene classification, DAVID (http://david.abcc.ncifcrf.gov/) was used.

For manuscripts utilizing custom algorithms or software that are central to the research but not yet described in published literature, software must be made available to editors/reviewers. We strongly encourage code deposition in a community repository (e.g. GitHub). See the Nature Research guidelines for submitting code & software for further information.

Data

Policy information about availability of data

All manuscripts must include a data availability statement. This statement should provide the following information, where applicable:

- Accession codes, unique identifiers, or web links for publicly available datasets
- A list of figures that have associated raw data
- A description of any restrictions on data availability

In page 71, the datasets generated and/or analyzed during the current study are also available from the corresponding authors upon reasonable request. The source data underlying Figs 1a-b, 1h, 2c, 3a-i, 4b-e, 5a-g, 6a-p and Supplementary Figs 1i, 3g, 4b-d, 4g, 5a-i, 6a-d, 6f, 7a-h, 7j-m, 7o-q and 8b-i are provided as a Source Data file.

Field-specific reporting

Please select the one below that is the best fit for your research. If you are not sure, read the appropriate sections before making your selection.

[x] Life sciences [ ] Behavioural & social sciences [ ] Ecological, evolutionary & environmental sciences

For a reference copy of the document with all sections, see nature.com/documents/nr-reporting-summary-flat.pdf

Life sciences study design

All studies must disclose on these points even when the disclosure is negative.

Sample size	In page 70-71, the numbers of samples used to result in statistically significant differences was calculated using G-power software with a significance level of 0.05 and a power of 0.8.
Data exclusions	We did not exclude the samples or animals except when the mice were dead.
Replication	All attempts at replication were successful.
Randomization	In page 62-63, we used block randomization method to allocate the samples/animals to experimental groups. This method is usually used to ensure a balance in sample size. For example, when two treatment group was tested, the block size is four (2 x 2= 4). Possible treatment allocations within each block are (1) AABB, (2) BBAA, (3) ABAB, (4) BABA, (5) ABBA, (6) BAAB. We selected the block size depended on the number of treatments. The block size was short enough to prevent imbalance, and long enough to prevent guessing allocation in trials. It was at least 2x number of treatments.
Blinding	In page 62-63 and 70-71, we were blinded some process of experimental progress such as data collection and data analysis. Experimenters were blinded to the identity of experimental groups until the end of data collection and analysis for at least one of the independent experiments.

Reporting for specific materials, systems and methods

We require information from authors about some types of materials, experimental systems and methods used in many studies. Here, indicate whether each material, system or method listed is relevant to your study. If you are not sure if a list item applies to your research, read the appropriate section before selecting a response.

Materials & experimental systems

n/a	Involved in the study	
[ ]	[x]	Antibodies
[ ]	[x]	Eukaryotic cell lines
[x]	[ ]	Palaeontology
[ ]	[x]	Animals and other organisms
[x]	[ ]	Human research participants
[x]	[ ]	Clinical data

Methods

n/a	Involved in the study	
[x]	[ ]	ChIP-seq
[ ]	[x]	Flow cytometry
[x]	[ ]	MRI-based neuroimaging

Antibodies

Antibodies used	We described the antibodies used in our study.<br>In page 57-58, the profiles of antibodies for immunofluorescence staining were as follows: 6E10 (mouse, 1:100, Signet, SIG39300), anti-20G10 (mouse, 1:1,000, provided by D.R. Howlett, GlaxoSmithKline, Harlow, Essex, UK) for A-beta 42, anti-G30 (rabbit, 1:1,000, provided by D.R. Howlett) for A-beta 40, SMA (mouse, 1:400, Clone 1A4, Sigma-Aldrich, A2547), AT8 (mouse, 1:500, Clone AT8, Thermo Fisher Scientific, MN1020), Caspase-3 (mouse, 1:200, Clone 31A1067, Novus Biologicals,

NB100-56708), ac-S565 (rabbit, 1:100), COX2 (mouse, 1:250, Clone COX 229, Thermo Fisher Scientific, 35-8200), Iba1 (rabbit, 1:500, Wako, 019-19741 and goat, Abcam, ab5076), NeuN (mouse, 1:500, Clone A60, Millipore, MAB377), GFAP (rabbit, 1:500, Dako, N1506 and chicken, 1:500, Abcam, ab4674), and Lamp1 (mouse, 1:200, Abcam, ab24170), COX2 (rabbit, 1:100, Abcam, ab15191), and CX3CR1 (mouse, 1:100, Clone SA011F11, BioLegend, 149008).

In page 58, the profiles of antibodies for western blotting were as follows: ac-S565 (rabbit, 1:500 and mouse, 1:500), COX2 (rabbit, 1:1,000, Abcam, ab15191), Synaptophysin (rabbit, 1:2000, Clone YE269, Abcam, ab32127), MAP2 (chicken, 1:10000, Abcam, ab5392), Synapsin 1 (rabbit, 1:1000, Synaptic systems, 106 103), PSD65 (mouse, 1:1000, Clone 6G6-1C9, Millipore, MAB1596), and beta-actin (mouse, 1:1,000, Clone C4, Santa Cruz, SC-47778).

In page 63-65, the profiles of antibodies for flow cytometry were as follows: mouse anti-CD45 PerCp Cy5.5 (Clone 30-F11; 550994; BD Bioscience), mouse anti-CD11b PE (Clone M1/70; 557397; BD Bioscience), mouse anti-CD86 PE-Cy7 (Clone GL1; 560582; BD Bioscience), mouse anti-CD206 APC (Clone C068C2; 141707; Bio legend), mouse anti-CD45 APC-Cy7 (Clone 30-F11; 557659; BD Bioscience), mouse anti-Gr-1 FITC (Clone RB6-8C5; 553126; BD Bioscience), mouse anti-MHCII PE (Clone M5/114; 557000; BD Bioscience), mouse anti-F4/80 APC (Clone BM8; 14-4801-82, Thermo Fisher Scientific), mouse anti-CD206 PE-Cy7 (Clone MR6F3; 25-2061-82; Thermo Fisher Scientific), mouse anti-Ly6C FITC (Clone AL-21; 553104; BD Bioscience), mouse anti-Ly6G APC-Cy7 (Clone RB6-8C5; 557661; BD Bioscience), mouse anti-CD11b APC (Clone M1/70; 553312; BD Bioscience), mouse anti-CD115 PE (Clone AFS98; 12-1152-82; Thermo Fisher Scientific), mouse anti-CD4 FITC (RM4-5; 553047; BD Bioscience), mouse anti-B220 PE (Clone RA3-6B2; 50-0452; Tonbo Bioscience), mouse anti-lineage biotin (130-090-858, Miltenyl Biotec), and anti-biotin streptavidin PB (S11222; Invitrogen).

In page 51-52, we described ac-S565 antibody generation. Polyclonal and monoclonal anti-acetyl COX2 S565 antibodies were generated using the acetylated COX2 peptide to immunize rabbits and mice (Genscrpit). Double affinity purification was performed using native and acetylated peptides sequentially using Sulfolink columns (Thermo Fisher Scientific). ac-S565 antibody was used for immunofluorescence staining (rabbit, 1:100) and western blotting (rabbit, 1:500 and mouse, 1:500).

Validation

Each primary antibody was validated using proper fluorophore controls and positive signal was evaluated versus an unstained sample. All antibodies have been previously published by our group (Nat Commun. 2018 Apr 16;9(1):1479.). All antibodies are from reputable vendors and come with validation statements on the manufacturer's website.

Eukaryotic cell lines

Policy information about cell lines

Cell line source(s)

In page 55-56, the adult immortalized human microglia cells-SV40 (T0251) were purchased from Applied Biologics Materials.

Authentication

Cell line authentiation was not performed.

Mycoplasma contamination

Cells were tested to confirm absence of mycoplasma contamination using MycoAlert PLUS Mycoplasma detection kit (Lonza, LT07).

Commonly misidentified lines
(See ICLAC register)

No commonly misidentified cell lines were used in this study.

Animals and other organisms

Policy information about studies involving animals; ARRIVE guidelines recommended for reporting animal research

Laboratory animals

In page 62-63, we used APP/PS1 mice (Transgenic mouse lines over-expressing the hAPP695swe (APP) and presenilin-1M146V (PS1) mutations) and 5xFAD mice (Jackson Labs, stock number. 34840). To examine possible therapeutic effect of N-AS and aspirin in APP/PS1 mice, the N-AS (30 mg kg-1, Sigma-Aldrich, 01912) was injected daily subcutaneously for 8 weeks to 7-mo-old APP/PS1 mice until the age of 9 months, and aspirin (2 mg kg-1, Sigma-Aldrich, A5376) was administrated orally to 7-mo-old APP/PS1 mice for 8 weeks. Data analysis was done in 1-, 3-, 5- or 9-month-old APP/PS1 mice. Also, to examine possible therapeutic effect of N-AS in 5xFAD mice, the N-AS (30 mg kg-1, Sigma-Aldrich, 01912) was injected daily subcutaneously for 4 weeks to 3-mo-old APP/PS1 mice until the age of 4 months. Data analysis was done in 4-mo-old 5xFAD mice. Because APP/PS1 and 5xFAD mice show sex differences in disease progression, we used only male mice. Mice were housed under standard conditions at a temperature of 22°C and a 12 h light/dark cycle with free access to tap water and food pellets.

Wild animals

No wild animals were used in the study.

Field-collected samples

No field-collected samples were used in the study.

Ethics oversight

Animal experiments were conducted in accordance with the protocols approved by the Institutional Animal Care and Use Committee (IACUC) of Kyungpook National University.

Note that full information on the approval of the study protocol must also be provided in the manuscript.

Flow Cytometry

Plots

Confirm that:

- [x] The axis labels state the marker and fluorochrome used (e.g. CD4-FITC).
- [x] The axis scales are clearly visible. Include numbers along axes only for bottom left plot of group (a 'group' is an analysis of identical markers).
- [x] All plots are contour plots with outliers or pseudocolor plots.
- [x] A numerical value for number of cells or percentage (with statistics) is provided.

Methodology

Sample preparation	In page 63-65, brains of WT, APP/PS1, and APP/PS1 treated with N-AS mice were dissected and immediately transferred in ice-cold HBSS (Gibco). After gentle mincing, the brain was digested in a HBSS solution containing collagenase P (0.2 mg ml-1, Roche), dispase II (0.8 mg ml-1, Roche), DNase I (0.01 mg ml-1, Roche), and collagenase A (0.3 mg ml-1, Roche) at 37 °C for 1h under gentle rocking. Digestion was stopped by adding FBS (Gibco) on ice. The supernatants were centrifuged at 250 g for 10 min at 4 ° C. The pellet was resuspended in 25 % BSA (Gibco)/PBS (Gibco) for myelin removal. Following a centrifugation step at 3,000 g for 30 min at 4 °C, the myelin containing supernatant was discarded. The cell pellets were then resuspended in 1 ml of HBSS and filtered through a 40 µm mesh, followed by a washing step in HBSS. The cell pellets were resuspended in 1 ml of red blood cell lysis buffer (BD Biosciences) and incubated for 10 min at RT for lysis of erythrocytes. Subsequently, 2 ml of MACs buffer (Miltenyl Biotec, 130-091-222) was added and centrifuged at 250 g for 10 min at 4 °C. And, blood of WT, APP/PS1, and APP/PS1 treated with N-AS mice was collected in sodium-heparin tube (367871, BD) by cardiac puncture. Red blood cells were lysed once for 10 min at 4 °C in 0.15 M NH4Cl (STEMCELL Technologies), washed once with PBS (Gibco), and counted using cell counter (Logos biosystems).The cells were stained with antibodies.
Instrument	The fluorescence data were acquired on a BD FACSAria III flow cytometer (BD).
Software	FACSDiva software (BD) was used to collec the data.<br>FlowJo analytical software (Tree Star, Inc.) was used to analyze the data.
Cell population abundance	The percentage of the relevant cell populations is reported in Fig. 4e, Fig. 5c-g, and Supplementary Fig. 5e-i.
Gating strategy	CD86+CD206-, CD86+CD206+, and CD86-CD206+ cells ; Cells were distinguished from debris by SSC/FSC, then Cells doubly positive for CD45 and CD11b were gated. The cell population were gated by CD206 and CD86.<br>CD45hiCD11b+Gr-1-F4/80+MHCIIhi and of CD45hiCD11b+Gr-1-F4/80+CD206hi cells; Cells were distinguished from debris by SSC/FSC, then signlets were selected on a FSC-H/FSC-W plot. The cell population were gated by DAPI (-). Then, the cell population were gated by CD45 (+), CD11b (+), Gr-1 (-). The cell population were gated by F4/80 (+) or MHCII (+).<br>CD45+CD11b+Ly6ChiLy6G- monocyte; Cells were distinguished from debris by SSC/FSC, then the cell population were gated by DAPI (-). Cells doubly positive for CD45 and CD11b were gated. The cell population were gated by Ly6C (+) and Ly6G (-).<br>CD45+CD11b+Gr-1+ neutrophil and CD45+Ly6G+Gr-1+ neutrophil; Cells were distinguished from debris by SSC/FSC, then the cell population were gated by DAPI (-). Cells positive for CD45 were gated. The cell population were gated by Gr-1 (+) and CD11b (+). The cell population were gated by Gr-1 (+) and Ly6G (+).<br>CD4+ T cell; Cells were distinguished from debris by SSC/FSC, then the cell population were gated by CD4 (+).<br>B220+ B cell; Cells were distinguished from debris by SSC/FSC, then the cell population were gated by B220 (+).<br>Lin-CD11b+F4/80hi macrophage; Cells were distinguished from debris by SSC/FSC, then the cell population were gated by Lin (-) and CD11b (+). The cell population were gated by F4/80 (+) and Ly6C (+).<br>CD11b+CD115+Ly6Chi and CD11b+CD115+Ly6Clow cells; Cells were distinguished from debris by SSC/FSC, then the cell population were gated by CD11b (+) and CD115(+). The cell population were gated by Ly6C (+) or Ly6C (-).<br>CD115lowLy6ChiLy6Ghi neutrophil; Cells were distinguished from debris by SSC/FSC, then the cell population were gated by CD115 (-) and Ly6C/Ly6G (+).

- [x] Tick this box to confirm that a figure exemplifying the gating strategy is provided in the Supplementary Information.
